# Conserved biophysical features of the Ca_V_2 presynaptic Ca^2+^ channel homologue from the early-diverging animal *Trichoplax adhaerens*

**DOI:** 10.1074/jbc.RA120.015725

**Published:** 2021-01-13

**Authors:** Julia Gauberg, Salsabil Abdallah, Wassim Elkhatib, Alicia N. Harracksingh, Thomas Piekut, Elise F. Stanley, Adriano Senatore

**Affiliations:** 1 Department of Biology, University of Toronto Mississauga, Mississauga, Ontario, Canada; 2 Laboratory of Synaptic Transmission, Krembil Research Institute, Toronto, Ontario, Canada

**Keywords:** Voltage-gated Ca2+ channels, CaV2 presynaptic Ca2+ channels, Trichoplax adhaerens, patch clamp electrophysiology, synapse evolution, Gβγ inhibition, pharmacology, ion channel, calcium channel, synapse, G protein, evolution, exocytosis, patch clamp, Gβγ-inhibition

## Abstract

The dominant role of Ca_V_2 voltage-gated calcium channels for driving neurotransmitter release is broadly conserved. Given the overlapping functional properties of Ca_V_2 and Ca_V_1 channels, and less so Ca_V_3 channels, it is unclear why there have not been major shifts toward dependence on other Ca_V_ channels for synaptic transmission. Here, we provide a structural and functional profile of the Ca_V_2 channel cloned from the early-diverging animal *Trichoplax adhaerens*, which lacks a nervous system but possesses single gene homologues for Ca_V_1–Ca_V_3 channels. Remarkably, the highly divergent channel possesses similar features as human Ca_V_2.1 and other Ca_V_2 channels, including high voltage–activated currents that are larger in external Ba^2+^ than in Ca^2+^; voltage-dependent kinetics of activation, inactivation, and deactivation; and bimodal recovery from inactivation. Altogether, the functional profile of *Trichoplax* Ca_V_2 suggests that the core features of presynaptic Ca_V_2 channels were established early during animal evolution, after Ca_V_1 and Ca_V_2 channels emerged via proposed gene duplication from an ancestral Ca_V_1/2 type channel. The *Trichoplax* channel was relatively insensitive to mammalian Ca_V_2 channel blockers ω-agatoxin-IVA and ω-conotoxin-GVIA and to metal cation blockers Cd^2+^ and Ni^2+^. Also absent was the capacity for voltage-dependent G-protein inhibition by co-expressed *Trichoplax* Gβγ subunits, which nevertheless inhibited the human Ca_V_2.1 channel, suggesting that this modulatory capacity evolved via changes in channel sequence/structure, and not G proteins. Last, the *Trichoplax* channel was immunolocalized in cells that express an endomorphin-like peptide implicated in cell signaling and locomotive behavior and other likely secretory cells, suggesting contributions to regulated exocytosis.

Voltage-gated Ca^2+^ (Ca_V_) channels serve essential functions in excitable cells, imparted by their capacity to translate electrical signals carried by Na^+^ and K^+^ channels, into cytoplasmic Ca^2+^ signals (1). For example, Ca_V_ channels couple membrane excitation with fusion of presynaptic vesicles, muscle contraction, alterations in nuclear gene expression, and regulation of ciliary beating (2, 3). Ca_V_ channels belong to a large family of pore-loop (P-loop) channels that includes voltage-gated Na^+^ (Na_V_) channels and K^+^ (K_V_) channels (4), named after their four extracellular loop structures that come together in the pore to form the ion selectivity filter, a motif uniquely configured in different channels for selecting Ca^2+^, Na^+^, or K^+^ ions (5). Humans and related animals possess three types of Ca_V_ channels, broadly classified as high and low voltage–activated, the former requiring strong depolarization for activation (*i.e.* Ca_V_1 or L-type channels and Ca_V_2 or N-, P-/Q-, and R-type channels) and the latter requiring only mild, sub-threshold depolarization (*i.e.* Ca_V_3 or T-type channels) (6). Phylogenomic studies have established that most animals possess single gene copies of Ca_V_1–Ca_V_3 channels, whereas gene duplications in vertebrates gave rise to four Ca_V_1 channels (Ca_V_1.1–Ca_V_1.4), three Ca_V_2 channels (Ca_V_2.1–Ca_V_2.3), and three Ca_V_3 channels (Ca_V_3.1–Ca_V_3.3) (3, 4, 7, 8, 9, 10, 11). Teleosts have had a further duplication of Ca_V_ channel genes, with species like *Danio rerio* having seven Ca_V_1, six Ca_V_2, and five Ca_V_3 genes (12). Independently, the cnidarians (*e.g.* jellyfish) duplicated Ca_V_2 and Ca_V_3 channel genes, resulting in a repertoire of a single Ca_V_1 channel, three Ca_V_2 channels, and two Ca_V_3 channels. The earliest diverging animal lineages possess only Ca_V_2 channels (ctenophores), Ca_V_1 channels (sponges), or an evolutionary precursor of Ca_V_1 and Ca_V_2 channels, dubbed Ca_V_1/2 channels (sponges) (3, 8, 10). The most early-diverging animals to possess all three Ca_V_ channel types (*i.e.* Ca_V_1–Ca_V_3) are the placozoans (3, 8, 10), a phylum of simple seawater animals that includes the species *Trichoplax adhaerens* and *Hoilungia hongkongensis* (13, 14). A unique feature of placozoans is that they lack neurons, synapses, and muscle (15, 16) and yet bear distinct cell types whose activity is coordinated for the purpose of motile behaviors such as feeding (17, 18), chemotaxis (19, 20, 21), phototaxis (20), and gravitaxis (22). Notably, despite lacking synapses, increasing evidence suggests that cellular communication in placozoans likely occurs in a protosynaptic manner, where regulated secretion of signaling molecules, such as neuropeptides and small-molecule transmitters, targets membrane receptors on other cells to exert an effect (18, 21, 23, 24).

In addition to their distinct voltages of activation, Ca_V_ channels are distinguished by their differential association with accessory Ca_V_β and Ca_V_α_2_δ subunits, which are essential for the proper membrane expression and function of Ca_V_1 and Ca_V_2, but not Ca_V_3 channels (2, 6). Furthermore, although their cellular functions overlap in certain contexts, there are several functions for which the different channels have specialized, observed nearly ubiquitously in animals ranging from humans to fruit flies to nematode worms (2, 3, 25). For example, endowed by their broadly conserved low activation voltages, Ca_V_3 channels tend to regulate membrane excitability in neurons and muscle, often in the context of rhythmic excitation, or to boost sub-threshold excitation as occurs in neuron dendrites (26, 27, 28, 29, 30, 31, 32, 33, 34). Instead, stronger depolarizing events, such as the action potential, activate Ca_V_2 channels, which are the major drivers of fast, synchronous membrane fusion of synaptic vesicles at the nerve terminal (35, 36, 37, 38, 39, 40, 41). Similarly, high voltage activation of post-synaptic Ca_V_1 channels in muscles and neurons drives contraction and changes in nuclear gene expression, respectively (2, 11, 33, 42, 43, 44, 45, 46, 47, 48). Indeed, given the considerable overlap in biophysical, ion-conducting properties of Ca_V_1 and Ca_V_2 channels, it is unclear why they have generally persisted in their unique respective post- and presynaptic functions.

Previously, we documented that the Ca_V_2 channel from the placozoan *T. adhaerens* lacks an acidic C-terminal amino acid motif proposed to be critical for interactions with presynaptic scaffolding proteins, such as Mint and RIM, and broadly conserved in animals with synapses, such as chordates, arthropods, nematodes, and cnidarians (10). Ca_V_1 channels also bear deeply conserved C-terminal motifs for interactions with post-synaptic proteins like Shank and Erbin (10). This suggests that a key evolutionary adaptation toward the specialization of Ca_V_1 and Ca_V_2 channels for distinct post- and presynaptic functions might have involved differential incorporation into protein complexes that would control trafficking and subcellular localization. Following the proposed Ca_V_1/Ca_V_2 split (8, 10), the two channel types might have also evolved biophysical features that distinguished them from each other. In the context of fast presynaptic exocytosis, ancestral Ca_V_2 channels might thus have borne unique biophysical features that made them particularly well-suited for this role. Given that placozoans lack synapses but are the most early-diverging animals to possess both Ca_V_1 and Ca_V_2 channels, they present an opportunity to address this question. Here, we sought to explore whether the Ca_V_2 channel from *T. adhaerens* exhibits biophysical features consistent with those of the major presynaptic Ca_V_2 channel isotype from humans, Ca_V_2.1. Cloning and *in vitro* expression of the *Trichoplax* Ca_V_2 channel, coupled with whole-cell patch-clamp electrophysiology, allowed us to compare its ion-conducting properties with those of human Ca_V_2.1 (49). Remarkably, despite roughly 600 million years of divergence, the *Trichoplax* channel exhibited functional features similar to those of the human channel, and its biophysical properties differed from those of the previously cloned *Trichoplax* low voltage–activated Ca_V_3 channel (28). Altogether, the work provides some important insights into the core features of synaptic Ca_V_2 channels, contributing to our understanding of the evolution of Ca_V_ channel function in animals.

## Results

### Cloning of a Ca_V_2 calcium channel homologue from T. adhaerens

**Figure 1:**
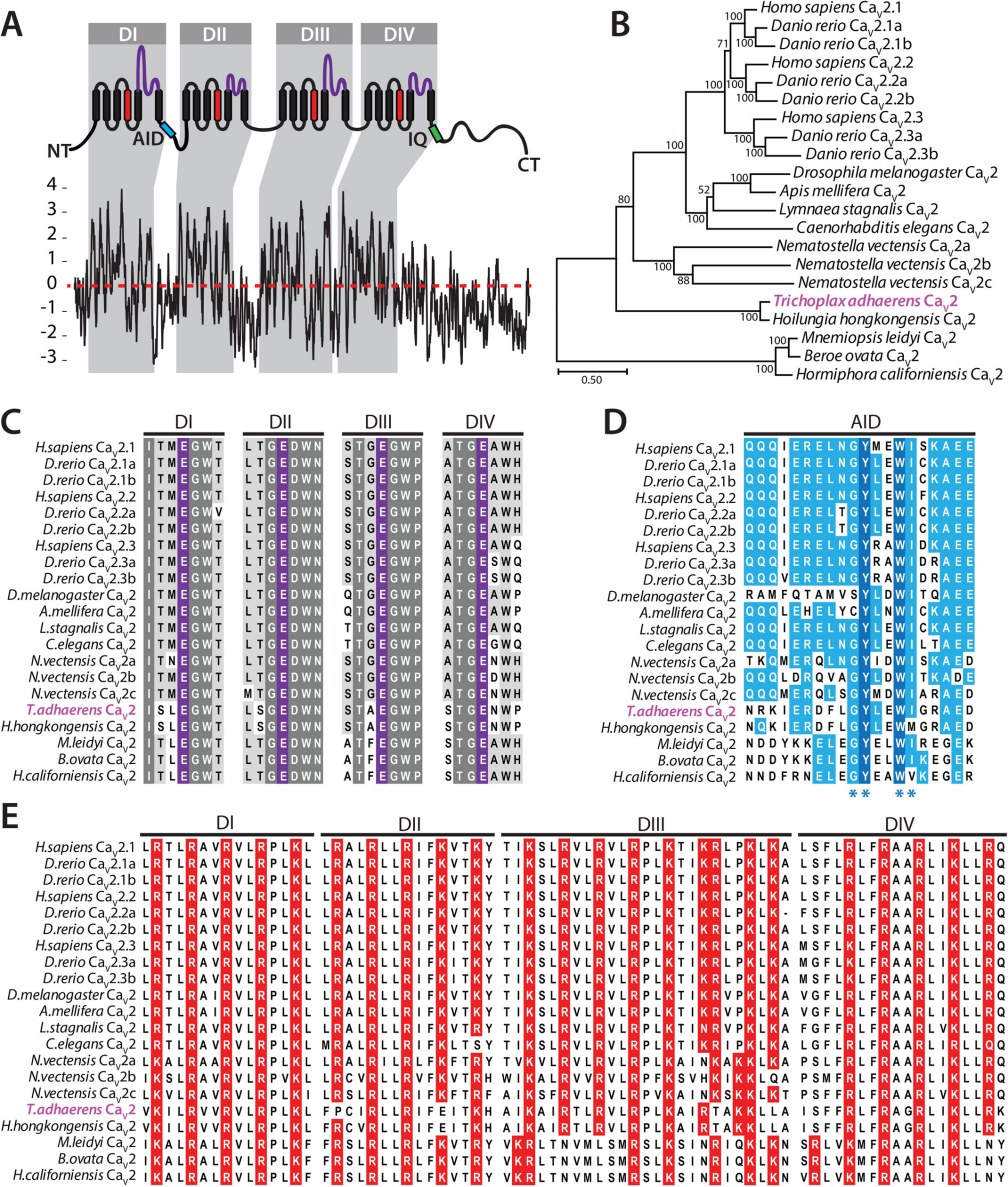
**Conserved structural features of the *Trichoplax* Ca_V_2 channel.***A*, Kyte–Doolittle plot of the TCa_V_2 protein sequence, revealing conserved hydrophobic peaks consistent with S1–S6 transmembrane segments arranged in four repeat domains (*DI–DIV*), separated by hydrophilic cytoplasmic linkers and N and C termini. The illustration *above* the plot denotes conserved features of Ca_V_2 channels, including the positively charged S4 helices that make up the voltage sensors (*red*), the pore loops that make up the selectivity filter (*purple*), the AID required for interactions with the Ca_V_β accessory subunit (*cyan*), and the IQ region required for interactions with the Ca^2+^ sensor protein calmodulin (*green*). *B*, maximum likelihood phylogenetic tree of various Ca_V_2 channel proteins, revealing the sister relationship of placozoan homologues with those from cnidarians and bilaterians. Consistent with the expected species phylogeny, the Ca_V_2 channel homologues from ctenophores are the most early-diverging Ca_V_2 channels. Bootstrap values for 1,000 ultrafast replicates are indicated at nodes, and branch lengths correspond to the *bar* on the *bottom left* indicating the unit of 0.5 substitutions per site. *C*, protein alignment of DI-IV pore-loop regions of various Ca_V_2 channels, revealing complete conservation of the four-glutamate (EEEE) selectivity filter motif. *D*, protein alignment of the AID, revealing that the TCa_V_2 AID bears a conserved glycine-tyrosine-*X*-*X*-tryptophan-isoleucine (GY-WI) amino acid motif essential for the Ca_V_β interaction (*blue asterisks*). *E*, alignment of the DI-IV S4 helices bearing repeating positively charged lysine and arginine residues critical for voltage sensing.

Previously, we identified a single putative *Trichoplax* Ca_V_2 (TCa_V_2) channel transcript, bearing a complete protein-coding sequence, in a whole-animal mRNA transcriptome (10, 50). The TCa_V_2 channel ORF was verified in triplicate via cloning of the corresponding cDNA from whole-animal total RNA, producing a consensus sequence encoding a 2,093-amino acid protein with a predicted mass of ∼240 kDa (GenBank^TM^ accession number MT506972). A Kyte-Doolittle hydrophobicity plot of the protein sequence showed hydrophobic peaks consistent with four repeat domains (DI–DIV), each with six transmembrane α helices (also known as segments 1–4 or S1–S4), and a long cytoplasmic C terminus (Fig. 1*A*). A maximum likelihood protein phylogeny of various Ca_V_2 channels exhibited complete lineage sorting with respect to the leading metazoan phylogeny (51), with TCa_V_2 and the Ca_V_2 channel homologue from fellow placozoan *H. hongkongensis* forming a sister relationship with cnidarian and bilaterian Ca_V_2 channels (Fig. 1*B*) and homologues from ctenophores forming the most distant clade of Ca_V_2 channels. As reported previously, placozoans are the most early-diverging animals to possess all three types of metazoan voltage-gated calcium channels (Ca_V_1–Ca_V_3), unlike ctenophores and sponges that lack Ca_V_3 (both phyla) and either Ca_V_1 (ctenophores and most sponges) or Ca_V_2 (sponges) channel homologues (8, 10, 52).

The TCa_V_2 channel bears a highly conserved Ca^2+^ ion selectivity filter motif of high voltage–activated Ca_V_1 and Ca_V_2 channels, made up of four negatively charged glutamate residues (*i.e.* EEEE motif) located within corresponding P-loop structures from each domain (Fig. 1, *A* and C). Also conserved is the α-interacting domain (AID), located within the cytosolic DI-DII linker; an α-helical structure unique to Ca_V_1 and Ca_V_2 channels that projects into the cytoplasm from the DI S6 helix and interacts with the cytoplasmic Ca_V_β subunit (Fig. 1*D*). It is notable that placozoan and ctenophore Ca_V_2 AID sequences retain conserved glycine-tyrosine-*X*-*X*-tryptophan-isoleucine (GY-WI) amino acid motifs that are essential for the Ca_V_β interaction (53), where mutations lead to disrupted modulation by Ca_V_β (54, 55). However, placozoan and ctenophore AIDs lack the signature glutamine triplet (QQQ) motif found in most other Ca_V_2 and Ca_V_1 channels. Accordingly, the *Trichoplax* genome encodes a single Ca_V_β gene, as well as three Ca_V_α_2_δ genes (14, 56), and these are known to be expressed at the RNA level (3).

Like other Ca_V_2 channels, the S4 α helices (also known as S4 segments) of TCa_V_2 bear repeating positively charged lysine and/or arginine residues critical for voltage sensing (Fig. 1*E*) (57). Among the channels analyzed, S4 segments in DI and DIV generally show the strongest conservation, with the exception of ctenophore Ca_V_2 channels that have shifted cationic charges in DIV toward the extracellular end of the S4 helix. Instead, S4 segments from DII and DIII are more variable for TCa_V_2 and other early-diverging homologues compared with bilaterian channels, notable because, at least for Ca_V_1.2 channels, these particular segments contribute disproportionately toward voltage sensing and channel activation (58). In DII, *Trichoplax* Ca_V_2 also differs from other Ca_V_2 channels, including *H. hongkongensis* Ca_V_2, with one less cationic charge due to an arginine to proline substitution. Despite some differences, the *Trichoplax* Ca_V_2 channel bears the core amino acid signatures required for gating the channel pore in response to changes in membrane voltage. This includes the highly conserved glutamate and aspartate residues located in S2 and S3 helices that counterbalance the positively charged arginine/lysine residues of S4 helices within each domain at rest and during channel activation, when S4 helices slide outward from the cell membrane upon depolarization (57) (Fig. S1).

Last, we explored the conservation of motifs for association with the Ca^2+^ sensor protein calmodulin (CaM), which dynamically interacts with Ca_V_1 and Ca_V_2 channels to modulate their activity in response to changes in cytoplasmic Ca^2+^ concentration and for the purpose of intracellular Ca^2+^ signaling (59). Ca^2+^-dependent regulation of Ca_V_ channels is likely ancient, observed in extant paramecia (60). Furthermore, the core C-terminal binding sites for CaM, known as pre-IQ and IQ motifs, are thought to have been present in the primordial ancestor of four-domain P-loop channels that gave rise to metazoan Ca_V_1/2, Ca_V_1, Ca_V_2, Ca_V_3, Na_V,_ and NALCN channels (4). A protein alignment of various Ca_V_2 channels reveals considerable conservation of amino acid sequence within the pre-IQ and IQ domains (Fig. S2), which contrasts with other cytoplasmic regions that tend to be highly divergent among distant Ca_V_ homologues (10). *Trichoplax* and *Hoilungia* Ca_V_2 channel IQ domains bear key amino acids for interacting with CaM, including an invariable isoleucine comprising the namesake IQ motif with consensus sequence (I/L/V)Q*XX*R*XXXX*(R/K) (61). Also conserved is an isoleucine six residues upstream of the IQ moiety and a doublet of tyrosine (YY) residues just downstream. In crystal structures of Ca_V_2.1 and Ca_V_2.3, the isoleucine residue is found anchored within a hydrophobic pocket of the N-lobe (N terminus) of CaM, and the tyrosine residues embed within the C-lobe (C terminus; Fig. S2) (62). About 35 residues upstream of the pre-IQ motif, Ca_V_1, and Ca_V_2 channels possess putative EF-hand Ca^2+^-binding motifs that are structurally indispensable for Ca^2+^/CaM-dependent regulation, independent of their capacity to bind Ca^2+^ (63). We note here that placozoan as well as invertebrate Ca_V_2 channels bear conserved EF-hand structures with amino acids capable of coordinating Ca^2+^ ions (Fig. S1). Conversely, the calmodulin-binding domain downstream of the IQ motif, reported for Ca_V_2.1 channels (64), is not immediately evident in the sequences of invertebrate Ca_V_2 channels (Fig. S1).

### TCa_V_2 expresses in vitro and is endogenously expressed in cells located around the periphery of the animal

**Figure 2:**
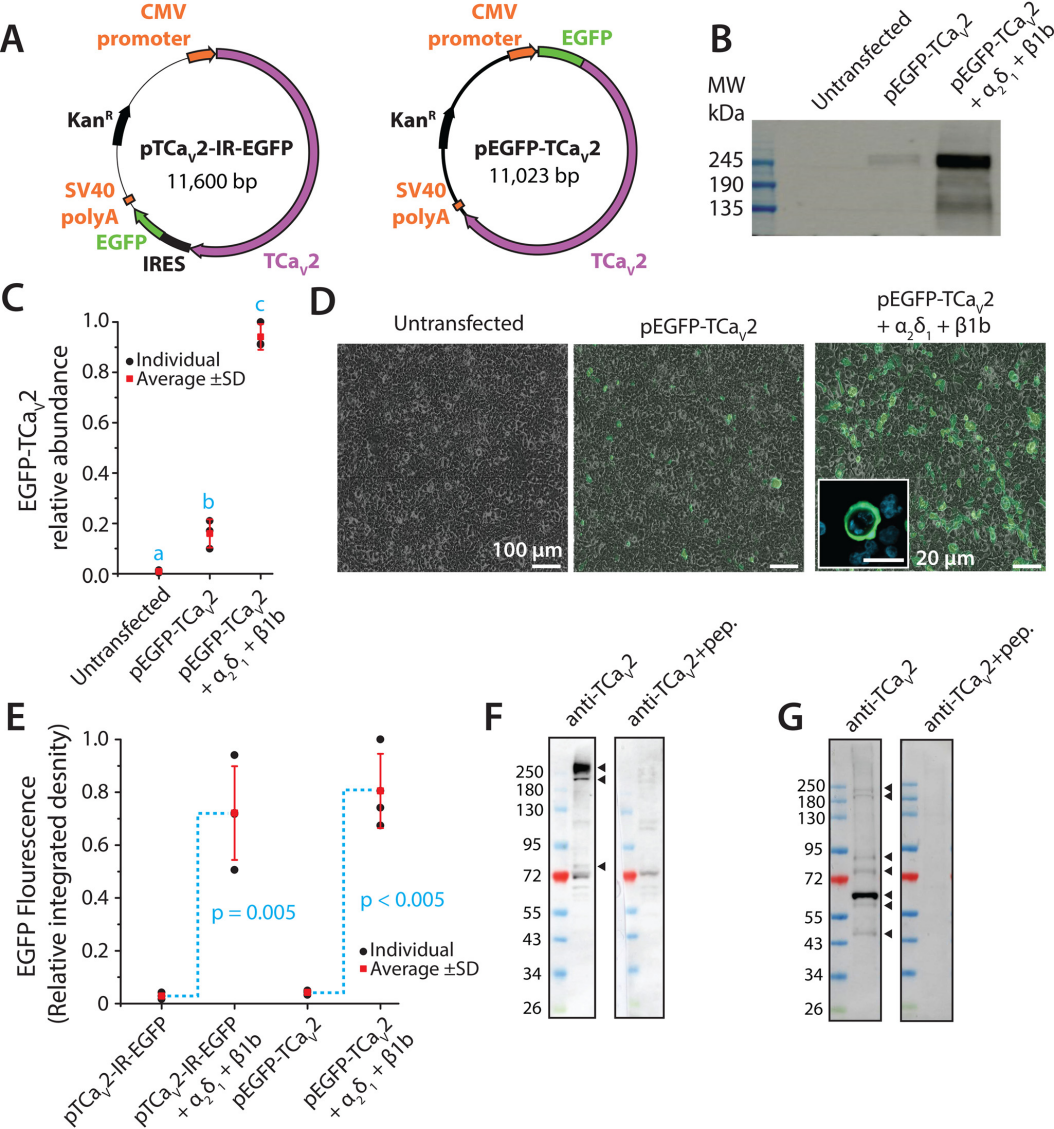
**TCa_V_2 is expressed as a full-length protein *in vitro* and *in vivo*.***A*, plasmid maps of pTCa_V_2-IR-EGFP and pEGFP-TCa_V_2 plasmid vectors for heterologous expression of the TCa_V_2 channel protein in mammalian cells. Whereas the pEGFP-TCa_V_2 vector expresses TCa_V_2 with an N-terminal EGFP fusion, pTCa_V_2-IR-EGFP permits expression of TCa_V_2 separately from EGFP. *B*, Western blotting of protein lysates from transfected HEK-293T cells with polyclonal anti-GFP antibodies reveals a band of about ∼270 kDa, consistent with the expected sum molecular weight of the TCa_V_2 plus the EGFP proteins. No such band is evident in untransfected cells, and its intensity is dramatically increased upon co-expression with the rat Ca_V_β_1_b and Ca_V_α_2_δ_1_ accessory subunits. *C*, quantification of band intensity on triplicate Western blots, relative to corresponding total protein on lanes of Coomassie-stained gels. All average values ± S.D. (*error bars*) are expressed relative to the maximal value across all experiments. *Lowercase letters* denote significant differences using a Holm–Sidak test (*p* < 0.006) after one-way ANOVA (*p* < 0.001, *F* = 387.6; Table S1). *D*, overlaid transmitted light and fluorescence images of HEK-293T cells depicting a dramatic increase in EGFP-TCa_V_2 fusion protein expression upon co-transfection with the rat Ca_V_β_1_b and Ca_V_α_2_δ_1_ accessory subunits. *Inset*, confocal image of a positively transfected HEK-293T cell expressing the EGFP-TCa_V_2 protein, with EGFP fluorescence visible in regions outside of the nucleus (stained with DAPI, *cyan*), consistent with endomembrane and cell membrane localization. *Scale bar*, 100 μm (*three larger panels*) and 20 μm (*inset*)). *E*, quantification of average pTCa_V_2-IR-EGFP and pEGFP-TCa_V_2 fluorescence intensity ± S.D. in triplicate micrographs of separately transfected HEK-293T cells is consistent with Western blotting results, where co-expression of the Ca_V_β_1_b and Ca_V_α_2_δ_1_ subunits dramatically increases protein expression of the EGFP-TCa_V_2 fusion protein, and EGFP expressed from the bicistronic vector.. Integrative density values were standardized to the maximal value across all experiments. Denoted *p* values for mean comparisons were generated using two-tailed tests. *F*, Western blotting of TCa_V_2 expressed in HEK-293T cells from the bicistronic pIRES2-EGFP vector, using rabbit polyclonal anti-TCa_V_2 antibodies directed against 142 amino acids in the II-III linker of the channel protein reveals strong ectopic expression of the *Trichoplax* calcium channel when co-expressed with rat Ca_V_β_1_b and Ca_V_α_2_δ_1_. At least three bands are visible on the blots, with molecular masses of about 250, 220, and 85 kDa, that disappeared after preincubation of the anti-TCa_V_2 antibodies with a recombinant epitope peptide. *G*, Western blotting of *Trichoplax* whole-animal protein lysates using the anti-TCa_V_2 polyclonal antibodies revealed numerous bands with molecular masses of about 240, 200, 90, 75, 63, 60, and 50 kDa, all of which disappeared after preincubation with recombinant blocking peptide.

The cDNA of TCa_V_2 was cloned into the mammalian expression plasmids pIRES2-EGFP and pEGFP-C1, producing corresponding TCa_V_2 protein expression vectors pTCa_V_2-IR-EGFP and pEGFP-TCa_V_2 (Fig. 2*A*). Transfection of pTCa_V_2-IR-EGFP into HEK-293T cells permits bicistronic expression of the channel separately from enhanced GFP (EGFP), whereas pEGFP-TCa_V_2 expresses TCa_V_2 tagged with EGFP at its N terminus (Fig. 2*A*). Using a commercial monoclonal anti-GFP antibody, the full-length EGFP-TCa_V_2 fusion protein could be detected in protein lysates of HEK-293T cells transfected with pEGFP-TCa_V_2 as a band with an estimated molecular mass of ∼270 kDa (Fig. 2*B*). This corresponds to expected sum molecular weight of EGFP (28.9 kDa) plus TCa_V_2 (239.5 kDa). Like the Ca_V_2 channel cloned from the snail *Lymnaea stagnalis* (37), efficient *in vitro* expression of TCa_V_2 in HEK cells required co-transfection with vectors encoding mammalian Ca_V_2 channel accessory subunits Ca_V_β (*i.e.* rat Ca_V_β1b) and Ca_V_α_2_δ (*i.e.* rat Ca_V_α_2_δ_1_), in lieu of *Trichoplax* subunits that were not part of this study (Fig. 2, *B* and *C*). This was also evident in fluorescence microscopy images of transfected cells, where fluorescence intensity of EGFP-TCa_V_2 was significantly higher when the HEK cells were co-transfected with Ca_V_β1b and Ca_V_α_2_δ_1_ cDNAs (Fig. 2, *D* and *E*).

We raised rabbit polyclonal antibodies against a recombinant peptide of 142 amino acids corresponding to the TCa_V_2 cytoplasmic II-III linker (Fig. S1), and tested their efficacy by Western blotting for untagged TCa_V_2 protein heterologously expressed in HEK-293T cells with the pTCa_V_2-IR-EGFP vector (co-transfected with rat Ca_V_β1b and Ca_V_α_2_δ_1_; Fig. 2*F*). The antibodies labeled a band at around 250 kDa, which disappeared after preincubation of membranes with the corresponding recombinant epitope peptide. Bands of about 220 and 85 kDa were also present and disappeared with peptide preincubation. The same antibodies were then used to detect TCa_V_2 in protein lysates isolated from *Trichoplax* whole animals, labeling bands at ∼240, 200, 90, 75, 63, 60, and 50 kDa on a Western blot (Fig. 2*G*). Although it is possible that some of the smaller-molecular weight bands correspond to off-target proteins, we note that BLAST searching the epitope protein sequence against *Trichoplax* transcriptome (50) and genome (14, 56) sequences fails to produce significant hits outside of the Ca_V_2 channel. As observed with the HEK-293T lysates, all bands disappeared after peptide preincubation, suggesting that although present as a full-length protein, some of the TCa_V_2 channel protein within the lysates is fragmented. Altogether, it is apparent that TCa_V_2 is expressed as a full-length protein in HEK-293T cells when heterologously expressed *in vitro* and as a full-length endogenously expressed protein in *Trichoplax*.

**Figure 3:**
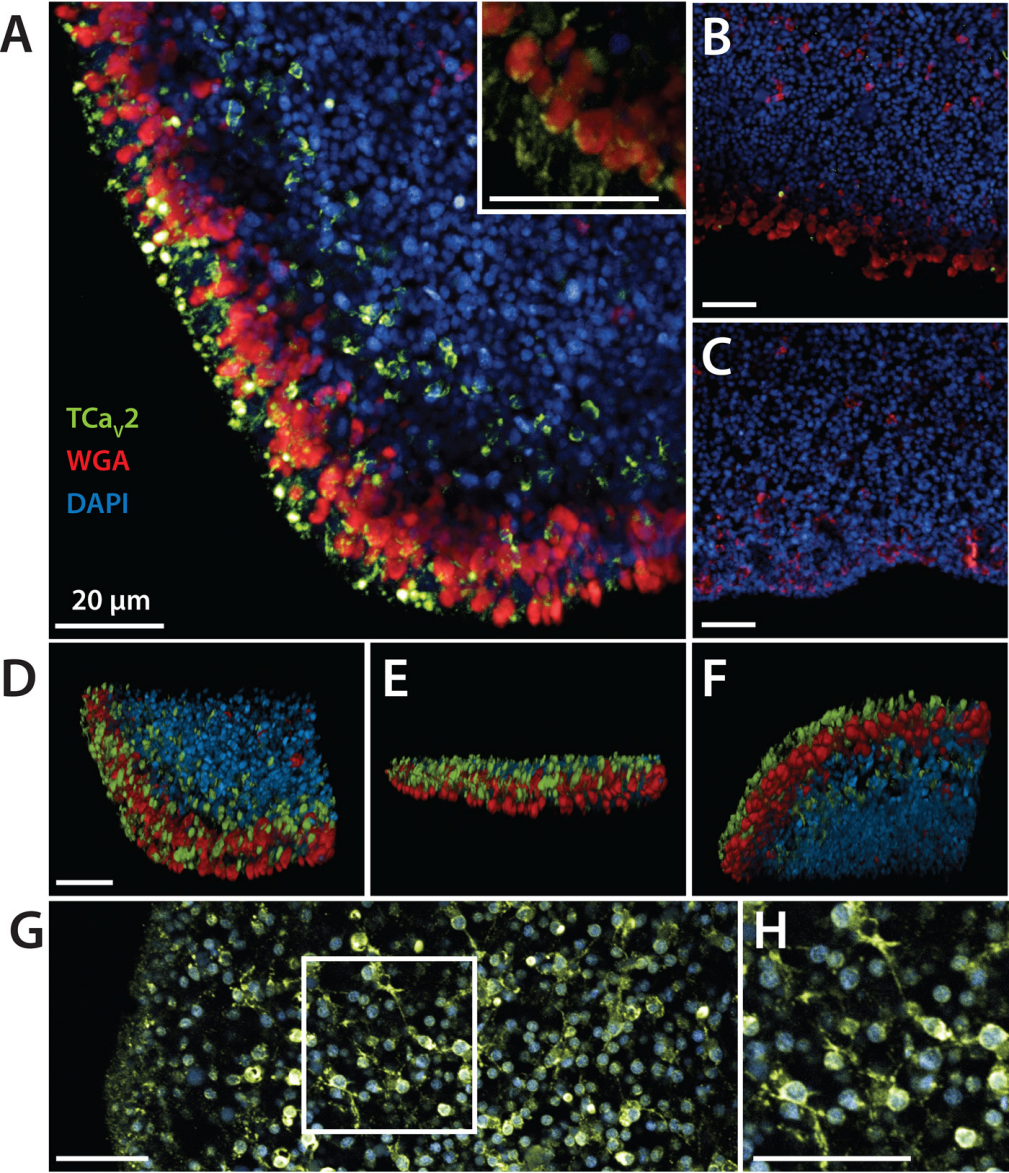
**TCa_V_2 is expressed in cells concentrated along the dorsal periphery of *Trichoplax*.***A*, maximum projection fluorescence micrograph of a *Trichoplax* animal stained *red* for type II gland cells using fluorophore-conjugated wheat germ agglutinin (WGA), *blue* for nuclei with DAPI, and immunolabeled with the rabbit anti-TCa_V_2 polyclonal antibody and a fluorescent anti-rabbit secondary antibody (*green*). *Inset*, co-expression of TCa_V_2 along the periphery of cells labeled with WGA. *B*, control experiment with preincubation of the TCa_V_2 antibody with the blocking peptide. *C*, control experiment lacking primary antibody. *D*, three-dimensional reconstruction of the micrograph stack shown in *A*, oriented from the top down, reveals expression of TCa_V_2 within a cluster of cells located along the outer edge of the dorsal epithelium. *E*, *side view* of the three-dimensional reconstruction, with the dorsal epithelium oriented at the *top*. *F*, *bottom view* of three-dimensional reconstruction reveals sparse labeling along the ventral epithelium. *G*, some preparations revealed TCa_V_2 staining within cells located in the interior of the animal, with filamentous structures consistent with fiber cells. *H*, enlarged view of the outlined region shown in panel *G*. *Scale bar*, 20 μm (all *panels*).

Applying the TCa_V_2 antibodies to whole-mount staining of fixed *Trichoplax* revealed expression around the periphery of the animal (Fig. 3*A*), in a region also labeled by fluorescent wheat germ agglutinin (WGA) that marks mucous-secreting type II gland cells, also referred to as mucocytes (23). At higher magnification, it is apparent that the TCa_V_2 protein is expressed within mucocytes, in small punctate regions adjacent to larger WGA-positive regions (Fig. 3*A*, *inset*). The latter likely represents clusters of mucous-containing vesicles labeled by WGA (23), demarking large cytoplasmic regions of separate mucocyte cells. Preincubation of the TCa_V_2 antibody with the blocking peptide, or staining in the absence of primary antibody, did not produce fluorescent signals above background levels, suggesting that the observed labeling was specific to TCa_V_2 (Fig. 3, *B* and *C*, respectively). Mucocytes, thought to constitutively secrete mucous for ciliary gliding/locomotion, also express the endomorphin-like peptide TaELP proposed to be subject to regulated secretion for the purpose of cell-cell signaling. Specifically, secreted TaELP is proposed to target receptors on ciliated ventral epithelial cells, pausing ciliary beating and hence causing locomotion to stop (18, 23). Three-dimensional rendering of the fluorescent images further reveals that although TCa_V_2 and WGA labeling overlap at the edge of the animal, TCa_V_2 is abundant along the dorsal epithelium, whereas mucocytes extend along the ventral epithelium (Fig. 3, *D–F*), as reported previously (23). Indeed, the dorsal staining observed for TCa_V_2 is consistent with staining patterns reported for two other *Trichoplax* regulatory peptides, SIFGamide and SITFamide, the former causing the animal to vigorously contract and “crinkle” and the latter slowing down ciliary locomotion (24). In some preparations, in addition to the labeling patterns noted above, the TCa_V_2 antibody labeled cells with branching filamentous structures consistent with fiber cells (Fig. 3, *G* and *H*), which are located between the dorsal and ventral epithelium and thought to be contractile in nature (15, 65, 66).

### TCa_V_2 conducts high voltage–activated Ca^2+^ currents in vitro that are similar to the human P/Q-type channel

**Figure 4:**
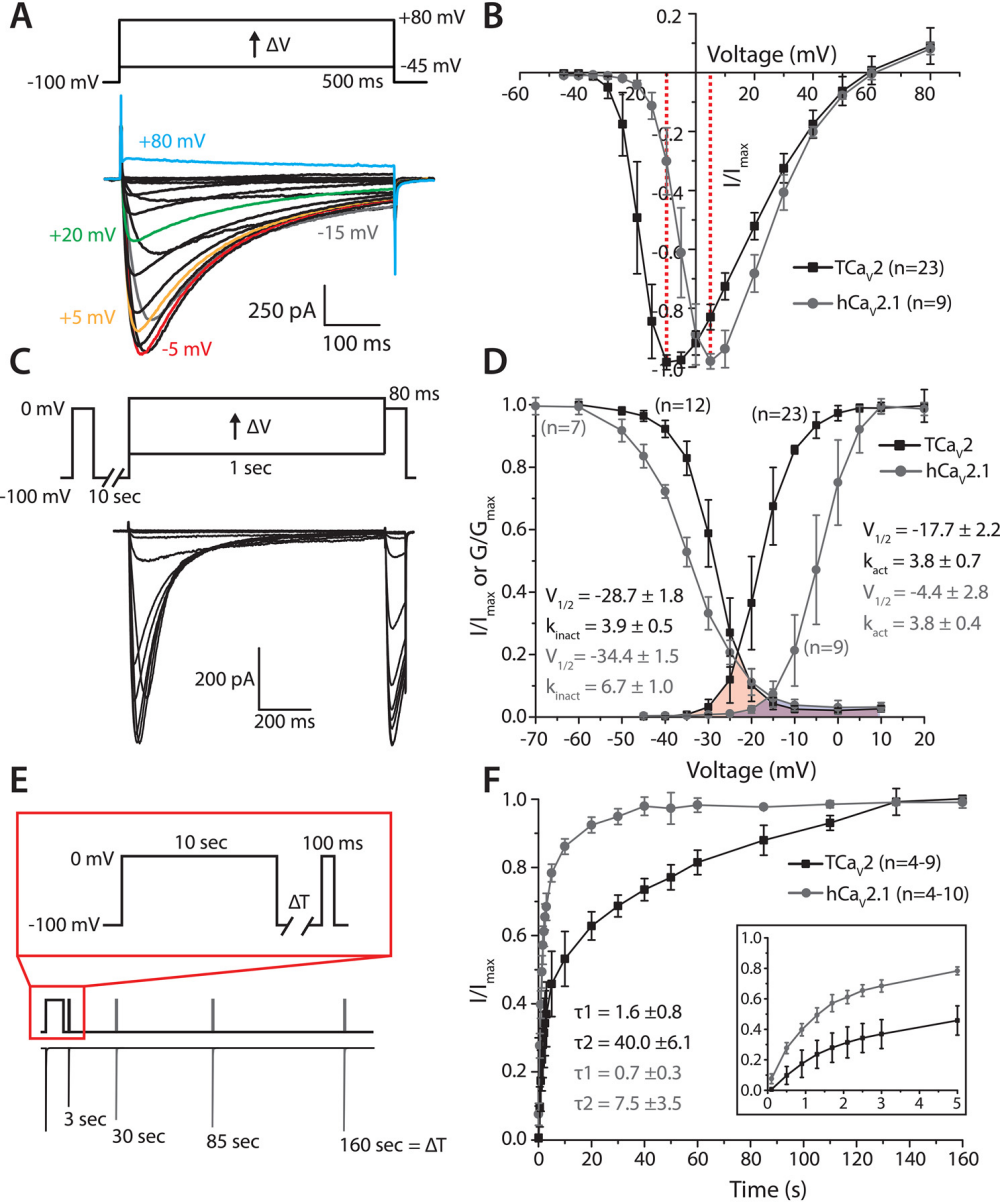
**TCa_V_2 produces robust voltage-gated Ca^2+^ currents *in vitro*.***A*, sample macroscopic current traces recorded via whole-cell patch voltage clamp of HEK-293T cells transfected with pTCa_V_2-IR-EGFP and rat Ca_V_β1b and Ca_V_α_2_δ_1_ subunits (*bottom*). The corresponding voltage-clamp protocol, with depolarizing voltage steps from −100 mV to various voltages, is depicted *above*. *B*, standardized average peak macroscopic current ± S.D. (*error bars*) plotted against corresponding voltage steps for TCa_V_2 and hCa_V_2.1 reveals a left-shifted maximal inward current for the *Trichoplax* channel. *C*, illustration of the voltage-clamp protocol used to assess inactivation of TCa_V_2 and hCa_V_2.1 (*top*). Peak amplitude of inward currents elicited by a test pulse following a 1-s pulse at various inactivating voltages is compared with that of a prepulse voltage step to 0 mV. Sample currents are shown *below*. *D*, plots of average inactivation ± S.D. and transformation of current-voltage plots into conductance plots reveal that, relative to hCa_V_2.1 (grey), the TCa_V_2 channel (black) is less sensitive to inactivation and more readily activated by small voltage steps. This results in a large window current at voltages where a subset of channels are not inactivated, and some become activated (*pink fill*). By comparison, the human channel has a much smaller window current component within the overlap between the inactivation and activation curves (*blue fill*). Average values for half-maximal activation and inactivation (*V*_½act_ and *V*_½inact_), plus *k*_activation_ and *k*_inactivation_ slope factors are depicted and were generated by fitting the activation and inactivation data with a Boltzmann function. *E*, voltage-clamp protocol used to assess recovery from inactivation of peak inward current after a 10-s inactivating pulse (*top*) and corresponding sample current traces recorded for the TCa_V_2 channel (*bottom*). *F*, plots of average recovery from inactivation ± S.D. of TCa_V_2 and hCa_V_2.1 reveal a slower recovery from inactivation for the *Trichoplax* channel. *Inset*, recovery data for the first 5 s, with inflections in the curves indicative of bimodal recovery from inactivation for both channels. Biexponential curve fitting over the data produced larger τ values for TCa_V_2 than hCa_V_2.1 (τ1 and τ2), especially for the slower recovery component.

Given that TCa_V_2 is expressed as a full-length protein in HEK-293T cells, we next sought to determine whether the recombinant channel could produce functional voltage-dependent Ca^2+^ currents *in vitro* using whole-cell patch-clamp electrophysiology. Voltage-clamp recordings of cells transfected with pTCa_V_2-IR-EGFP, along with expression vectors for rat Ca_V_β1b and Ca_V_α_2_δ_1_, revealed large-amplitude Ca^2+^ currents in 3 mm external Ca^2+^ solution that could be elicited by depolarization from −100 mV to between −45 and +80 mV (Fig. 4*A*). The amplitudes of inward macroscopic Ca^2+^ currents were quite variable (Fig. S3) and, in some cells, were greater than 2,000 pA. Thus, to prevent voltage errors in our recordings that can be caused by large amplitude currents, we only used cells with peak inward currents near and below 1,000 pA and patch pipettes with minimal access resistance (see “Experimental procedures”). When only the rat Ca_V_β1b and Ca_V_α_2_δ_1_ subunit cDNAs were transfected, no calcium currents could be recorded. In contrast to the low voltage–activated Ca_V_3 channel cloned from *Trichoplax* (28), TCa_V_2 activation required strong depolarization, with inward Ca^2+^ currents first appearing at voltage steps to –35 mV and maximal peak macroscopic current occurring at −10 mV (Fig. 4*B*). To provide a comparative context to the TCa_V_2 electrophysiology experiments, the cloned human P/Q-type (Ca_V_2.1) channel (49) was also expressed in HEK-293T cells using the same conditions and recording solutions. Although TCa_V_2 is a high voltage–activated channel like other Ca_V_2 channels, its voltage sensitivity is left-shifted compared with human Ca_V_2.1 (hCa_V_2.1), the latter having a maximal inward current at +5 mV (Fig. 4*B*). Transformation of peak inward currents into conductance values, a process that removes the effect of driving force and enables visualization of macroscopic conductance as the channel population responds to depolarization, reveals that TCa_V_2 achieves half-maximal activation (*V*_½_) at −17.7 ± 2.2 mV, compared with −4.4 ± 2.8 mV for hCa_V_2.1 (Fig. 4*D*). Despite this roughly 13-mV difference, the rate of activation of TCa_V_2 and hCa_V_2.1 in response to depolarization is similar, with respective conductance curve slope (*k*_activation_) values of 3.8 ± 0.7 and 3.8 ± 0.4 mV (not significantly different, two-tailed test *p* = 0.956). We also compared the inactivation properties of TCa_V_2 and hCa_V_2.1, which approximates the fraction of channels within a population available for activation at different values of resting membrane potential, albeit within a relatively transient time scale. Holding voltages ranging from −60 to +10 mV, held for 1 s, caused the amplitude of macroscopic Ca^2+^ currents elicited by a test pulse to 0 mV to gradually decline relative to a prepulse to 0 mV due to accumulating inactivation within the channel population (Fig. 4*C*). Plotting the ratio of maximal inactivated current amplitude *versus* test pulse current amplitude, as a function of the inactivating voltage, revealed an inactivation curve for TCa_V_2 with a *V*_½_ at −28.7 ± 1.8 mV and a slope (*k*_inactivation_) of 3.9 ± 0.5 mV (Fig. 4*D*). In contrast, hCa_V_2.1 exhibited a hyperpolarized shift in *V*_½_ of inactivation with a value of −34.4 ± 1.5 mV and also a slower rate of inactivation with a *k*_inactivation_ of 6.7 ± 1.0 mV (two-tailed 3.94 test *p* < 0.001). Notable is that the relatively right-shifted inactivation curve of TCa_V_2, coupled with its left-shifted activation curve, produces a substantial window current voltage range between −35 and −10 mV (Fig. 4*D*, *red fill*), much more prominent than observed for hCa_V_2.1 (Fig. 4*D*, *blue fill*). This feature is more commonly attributed to Ca_V_1 and Ca_V_3 channels and represents a range of resting voltages through which a subset of channels would remain open to conduct constitutive Ca^2+^ currents into the cell (67, 68).

A feature of Ca_V_ channels that determines their continued contribution to rises in intracellular Ca^2+^ during prolonged bouts of excitation (*e.g.* action potential burst firing) is their recovery from inactivation. Whereas a population of Ca_V_ channels with a fast recovery from inactivation can remain active throughout a train of action potentials, those with slow recovery tend to accumulate inactivation and hence contribute less Ca^2+^ influx toward the end of an action potential burst (69). Recovery from inactivation of TCa_V_2 and hCa_V_2.1 was assessed by determining the peak current that could be elicited by a step to 0 mV at different time intervals succeeding a 10-s inactivating pulse to 0 mV (Fig. 4*E*). Consistent with previous reports, the hCa_V_2.1 channel exhibited bimodal recovery from inactivation (69), with respective time constants for fast and slow components of the recovery process of 0.7 ± 0.3 s (τ1) and 7.5 ± 3.5 s (τ2) (Fig. 4*F*). Interestingly, the TCa_V_2 channel also exhibited bimodal recovery from inactivation, with a similar fast component (τ1 = 1.6 ± 0.8 s; *p* = 0.713) but a much slower slow component compared with hCa_V_2.1 (τ2 = 40.0 ± 6.1 s; *p* < 0.001 for Holm–Sidak test after two-way ANOVA with *p* < 0.001 and *F* ≥ 90.175 for all comparisons; Table S1). Altogether, the slower recovery from inactivation kinetics of TCa_V_2 is evident after 3 s of hyperpolarization following the inactivating pulse, where 68.4 ± 3.9% of hCa_V_2.1 channels had recovered, compared with only 36.9 ± 9.6% of TCa_V_2 channels (Fig. 4*F*, *inset*). Similarly, TCa_V_2 required much more time for full recovery from inactivation, at roughly 135 s (99.2 ± 3.8% recovery) compared with 40 s for hCa_V_2.1 (98 ± 2.7% recovery).

### The kinetic properties of TCa_V_2 macroscopic currents resemble those of hCa_V_2.1 in their voltage dependence

**Figure 5:**
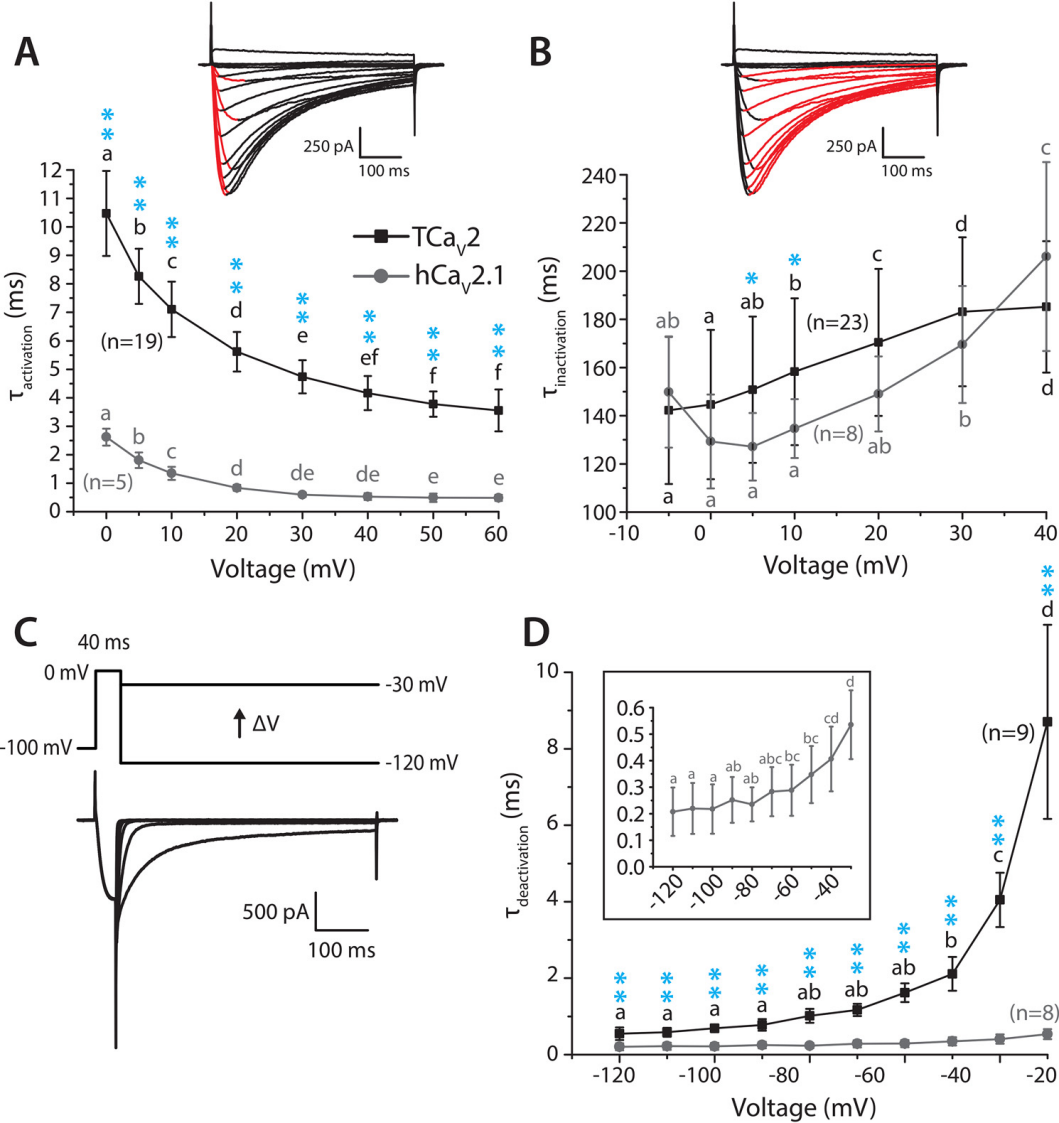
**Although generally slower, the kinetics of TCa_V_2 macroscopic currents resemble those of hCa_V_2.1 in their voltage dependence.***A*, plot of average τ values ± S.D. obtained by monoexponential curve fitting over the activation phase of macroscopic currents elicited by different depolarizing voltages. Changing τ_activation_ values for both TCa_V_2 and hCa_V_2.1 indicates accelerating activation with stronger depolarization. Nevertheless, activation of TCa_V_2 was significantly slower at all depolarizing voltages. *B*, plot of average τ values ± S.D. (*error bars*) obtained by monoexponential curve fitting over the inactivation phase of macroscopic currents elicited by different depolarizing voltages. Both TCa_V_2 and hCa_V_2.1 exhibit increasing τ_inactivation_ values with stronger depolarization. *C*, protocol used to assess deactivation kinetics, with hyperpolarizing pulses to varying voltages following a brief step to 0 mV (*top*). Sample tail current traces recorded for the TCa_V_2 channel are shown *below*. *D*, plot of average τ_deactivation_ values ± S.D. for TCa_V_2 and hCa_V_2.1 revealing accelerating deactivation upon stronger hyperpolarization for both channels. Across all voltages, the *Trichoplax* channel exhibits slower deactivation kinetics than hCa_V_2.1. *Letters above* the data points indicate statistically significant differences resulting from paired comparisons using a Holm–Sidak test after respective one-way repeated measures ANOVA for TCa_V_2 and hCa_V_2.1 τ values (*i.e.* where *p* < 0.01). Thus, plotted values bearing the same *letter* are not statistically different from each other. *Single* and *double cyan asterisks* denote respective post hoc Holm–Sidak *p* values of <0.05 and <0.001 for paired comparisons of τ values for TCa_V_2 and hCa_V_2.1 currents at different voltages, after two-way ANOVA (Table S1).

We compared the kinetic properties of TCa_V_2 and hCa_V_2.1 activation and inactivation by fitting monoexponential curves over the rise and decay phases of macroscopic currents, producing corresponding time constants (τ_activation_ and τ_inactivation_). Both channels exhibited accelerating activation kinetics with increasing depolarization, with roughly 2-fold decreases in τ_activation_ at +60 mV compared with 0 mV (Fig. 5*A*) (*p* < 0.001 and *F* ≥ 99.123 for one-way repeated measures ANOVAs for TCa_V_2 and hCa_V_2.1; Table S1). Nevertheless, activation of the *Trichoplax* channel was much slower than hCa_V_2.1, with respective τ_activation_ values of 10.5 ± 1.5 and 2.6 ± 0.3 ms at 0 mV, decreasing to 3.6 ± 0.7 and 0.5 ± 0.1 ms at +60 mV (*p* < 0.001 for Holm–Sidak test after two-way ANOVA; *p* < 0.001 and *F* ≥ 18.201 for all comparisons; Table S1). Kinetics of inactivation for both channels were also voltage-dependent, but in contrast to activation kinetics, τ_inactivation_ showed a general deceleration with increasing depolarization (Fig. 5*B*; *p* < 0.001 and *F* ≥ 14.301 for one-way repeated measures ANOVAs for TCa_V_2 and hCa_V_2.1; Table S1). Within the voltage range tested, hCa_V_2.1 showed first acceleration in inactivation kinetics from −5 to 0 mV, followed by deceleration toward +40 mV. This is in contrast to TCa_V_2, which exhibited faster inactivation kinetics at −5 mV and a continual voltage-dependent deceleration until +40 mV. Altogether, the trajectories of respective τ_inactivation_ curves over the tested voltage range were similar, with TCa_V_2 exhibiting slower inactivation kinetics than hCa_V_2.1 at the voltages of 5 and 10 mV (*p* < 0.05 for Holm–Sidak test after two-way ANOVA; *p* = 0.029 and *F* = 4.824 for variation due to channels, *p* < 0.001 and *F* = 12.942 for variation due to voltage, *p* = 0.063 and *F* = 2.036 for variation due to the interaction of channel and voltage; Table S1).

Another important factor that determines the amount of Ca^2+^ influx through Ca_V_ channels is their deactivation kinetics, which reflects how quickly open channels transition to a closed, activatable state upon membrane hyperpolarization. During action potential repolarization and hyperpolarization, Ca_V_ channels with slow deactivation remain open longer to conduct a surge of Ca^2+^ into the cytoplasm (facilitated by the increased driving force for inward Ca^2+^ flow at negative potentials), compared with fast deactivating channels that would quickly close (70). To compare the deactivation kinetics of TCa_V_2 and hCa_V_2.1, monoexponential curves were fitted over decaying macroscopic currents elicited through open channels upon hyperpolarization to between −120 and –30 mV (Fig. 5*C*). Similar to τ_inactivation_, τ_deactivation_ for both channels exhibited voltage-dependent deceleration, most striking for TCa_V_2 with τ_deactivation_ increasing from 0.5 ± 0.2 ms at −120 mV to 8.7 ± 2.5 ms at −30 mV, compared with 0.2 ± 0.1 ms at −120 mV to 0.5 ± 0.1 ms at –30 mV for hCa_V_2.1 (Fig. 5*D*). At the more depolarized voltages of −40 and −30 mV, there is a theoretical but perhaps small possibility that channel inactivation might be contributing to the current decay, especially for TCa_V_2, which undergoes marginal activation and considerable inactivation at these voltages (Fig. 4*D*). However, we note that the decaying currents were best fit with monoexponential functions, indicative of a single component process. At all voltages, TCa_V_2 deactivation was slower than hCa_V_2.1 deactivation (*p* < 0.001 for Holm–Sidak test after two-way ANOVA; *p* < 0.001 and *F* ≥ 6.842 for all comparisons; Table S1).

### TCa_V_2 resembles hCa_V_2.1 and other high voltage–activated calcium channels by conducting larger Ba^2+^ than Ca^2+^ currents

**Figure 6:**
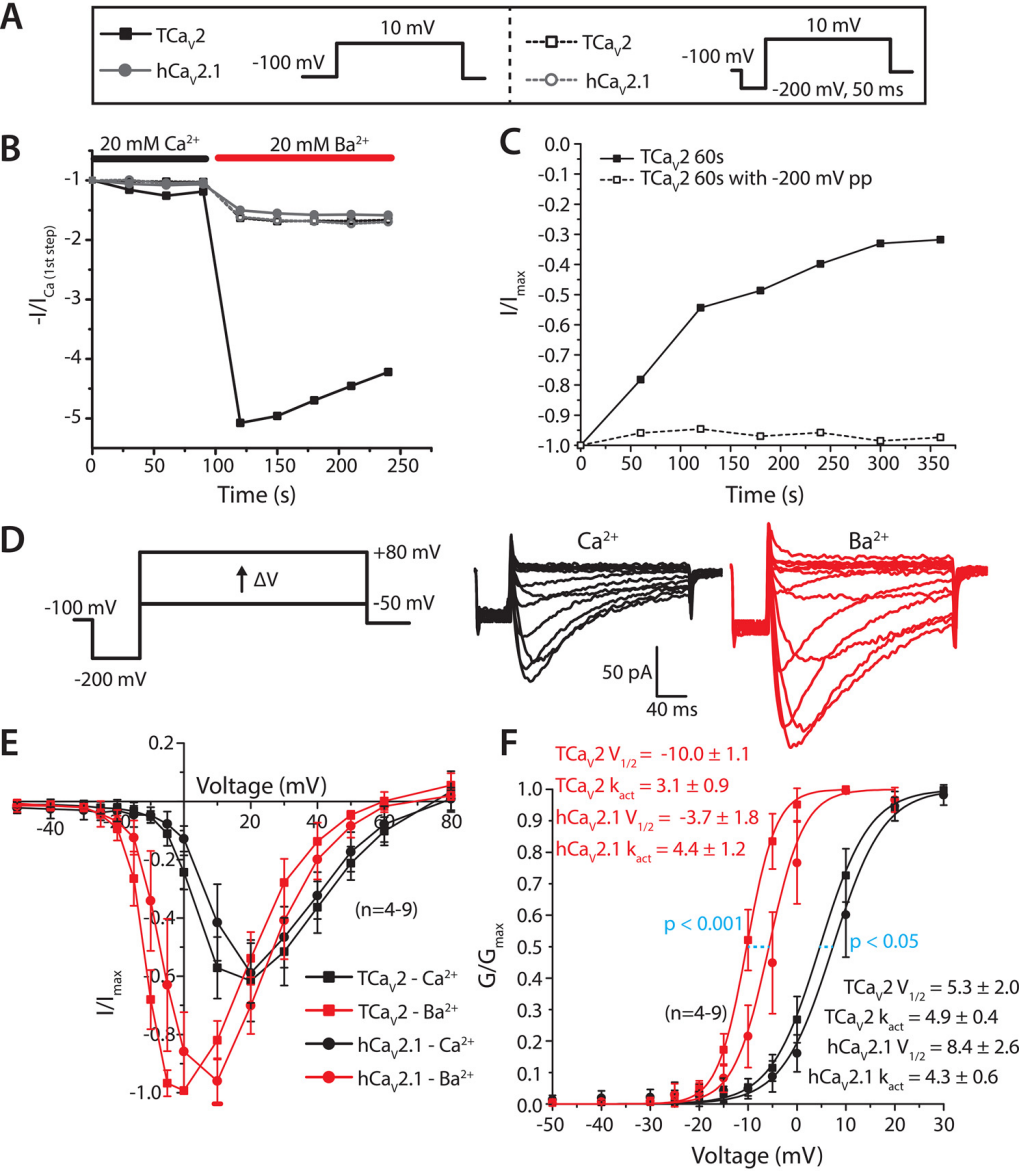
**TCa_V_2 resembles hCa_V_2.1 by conducting larger macroscopic Ba^2+^ than Ca^2+^ currents.***A*, voltage step protocols to record inward currents at 10 mV without (*left*) and with (*right*) a 200-mV, 50-ms prepulse. *B*, peak currents for TCa_V_2 and hCa_V_2.1 elicited by the protocols depicted in *A*, while perfusing 20 mm external Ba^2+^ or Ca^2+^ solutions, normalized against the peak amplitude of the first traces recorded in Ca^2+^. Without a prepulse, the Ba^2+^ currents through the TCa_V_2 are markedly larger and decay upon subsequent voltage steps. *C*, representative plot of relative peak TCa_V_2 inward currents elicited by the protocols in *A*, but with a longer interpulse interval of 60 s, indicates that the decaying currents in the presence of Ba^2+^ are not likely to be due to accumulated inactivation. *D*, illustration of the voltage-clamp protocol used to compare relative current-voltage properties of TCa_V_2 and hCa_V_2.1 while perfusing 20 mm external Ca^2+^ or Ba^2+^ (*left*). Sample Ca^2+^ and Ba^2+^ current traces recorded from a single cell expressing TCa_V_2 are shown on the *right*. *E*, current-voltage plots of TCa_V_2 and hCa_V_2.1 average peak inward current ± S.D. (*error bars*), relative to maximal Ba^2+^ current, reveal similar increases in current amplitude and leftward shifts in maximal inward current for both channels when Ba^2+^ is used as a charge carrier. *F*, transformation of the current-voltage plots in *E* reveals left-shifted activation curves in the presence of external Ba^2+^, particularly for TCa_V_2. Average values ± S.D. for *V*_½act_, *V*_½inact_, *k*_activation_, and *k*_inactivation_ slope factors were generated by fitting the activation data with a Boltzmann function. The reported *p* values in the plot are for Holm–Sidak comparisons of mean *V*_½_ values for current activation curves for TCa_V_2 and hCa_V_2.1 in Ca^2+^ and Ba^2+^ after two-way ANOVA (Table S1).

The nonphysiological cation Ba^2+^ is used as a surrogate for Ca^2+^ in electrophysiological recordings of Ca_V_ channels, likely for its tendency to produce large currents compared to other divalent cations, plus its ability to potently block K^+^ channel currents (71). Generally, high voltage–activated Ca_V_ channels such as L-type (Ca_V_1), N-type, and P/Q-type (Ca_V_2) conduct larger macroscopic Ba^2+^ than Ca^2+^ currents (71, 72, 73), whereas T-type (Ca_V_3) channels tend to vary in this respect between paralogous subtypes (*i.e.* Ca_V_3.1–Ca_V_3.3) and species (26, 27, 28, 29, 33, 74). We sought to compare the permeation properties of TCa_V_2 and hCa_V_2.1 for Ca^2+^ and Ba^2+^ ions. Voltage steps from –100 to +10 mV at 30-s intervals (Fig. 6*A*) produced stable TCa_V_2 macroscopic currents while perfusing 20 mm external Ca^2+^. Currents increased in amplitude ∼4.3-fold upon switching to 20 mm external Ba^2+^ (Fig. 6*B*). This is in contrast to hCa_V_2.1, which only showed a ∼1.6-fold increase when Ca^2+^ was replaced with Ba^2+^. Notably, the peak Ba^2+^ currents for TCa_V_2 decayed in amplitude with sequential voltage pulses, a rare phenomenon also reported for Ca_V_ channels recorded in the somatic membrane of mollusc neurons (75). This use-dependent decay prevented us from accurately recording current-voltage data for the channel for comparing Ba^2+^ *versus* Ca^2+^ permeation properties across a range of voltage steps. However, we noticed that the decay process could be stopped by preceding the depolarizing voltage steps with 50-ms hyperpolarizing prepulses to −200 mV, similar to what was reported for the decaying Ca_V_ channel Ba^2+^ currents recorded in snail neurons (75). With the prepulse, the increase in peak current for TCa_V_2 when switching from Ca^2+^ to Ba^2+^ was reduced to resemble that of the human channel, with only a ∼1.5-fold increase (Fig. 6*B*). We note that although the hyperpolarization step had an effect on TCa_V_2 channel Ba^2+^ currents, it did not affect its Ca^2+^ currents or the hCa_V_2.1 Ca^2+^ or Ba^2+^ currents, verified across a range of voltages (−50 to +80 mV, not shown). To further understand the decaying Ba^2+^ currents for TCa_V_2, we sought to rule out the anomalous mole fraction effect that can occur when perfusing solutions containing Ca^2+^ and Ba^2+^ over cells (76). Cells expressing TCa_V_2 channels were therefore placed into a bath containing only 20 mm Ba^2+^ solution. However, the decay process was still evident (Fig. 6*C*), ruling out the anomalous mole fraction effect. Furthermore, to rule out the possibility that Ba^2+^ enhances accumulation of inactivated channels, intersweep intervals were increased to 60 s. Under these conditions, peak Ba^2+^ currents once again exhibited a decay in amplitude in the absence of a hyperpolarizing prepulse, which was not observed when a hyperpolarizing prepulse was applied (Fig. 6*C*).

In light of these observations, we introduced a similar −200-mV prepulse to generate TCa_V_2 and hCa_V_2.1 current-voltage data while perfusing 20 mm Ca^2+^ or Ba^2+^ (Fig. 6*D*). We also selected cells with peak Ca^2+^ current amplitudes of roughly 100 pA, such that voltage errors did not arise when switching to Ba^2+^ due to larger current amplitudes. For both channels, plots of peak current at different voltages revealed similar increases in maximal inward current of roughly 1.6-fold in Ba^2+^ compared with Ca^2+^, with both channels reaching maximal current at +20 mV in the presence of Ca^2+^, shifting leftward to 0 and +10 mV, respectively, for TCa_V_2 and hCa_V_2.1 in the presence of Ba^2+^ (Fig. 6*E*). Notably, the overlapping IV data for TCa_V_2 and hCa_V_2.1 in 20 mm Ca^2+^, a condition that resembles the physiological Ca^2+^ concentration in seawater, contrast with our previous observations in 3 mm Ca^2+^, where peak TCa_V_2 currents were left-shifted relative to hCa_V_2.1 (Fig. 4*B*). Transformation of the current-voltage data to relative conductance plots revealed similar voltages of half-maximal activation in 20 mm Ca^2+^ for the two channels (TCa_V_2 *V*_½_ = 5.3 ± 2.0 mV; hCa_V_2.1 *V*_½_ = 8.4 ± 2.6 mV; Fig. 6*F*), although statistically significantly different from each other (*p* < 0.05 for Holm–Sidak test after two-way ANOVA; *p* < 0.001 and *F* ≥ 29.602 for variation due to ions and channels, and *p* = 0.079 and *F* = 3.435 for variation due to the interaction of ions and channels; Table S1). Activation curves were left-shifted in the presence of Ba^2+^ (TCa_V_2 *V*_½_ = −10.0 ± 1.1 mV; hCa_V_2.1 *V*_½_ = −3.7 ± 1.8 mV), more so for TCa_V_2 (*p* < 0.001 for Holm–Sidak test after two-way ANOVA; Table S1). Furthermore, *k*_activation_ slope values in Ba^2+^ were statistically significantly different between the two channels (*p* < 0.05 for Holm–Sidak test after two-way ANOVA; *p* = 0.310 and *F* = 1.086 for variation due to ion type, *p* < 0.05 and *F* ≥ 4.716 for variation due to channels as well as ion and channel interactions; Table S1), although they were not in Ca^2+^.

### TCa_V_2 exhibits low sensitivity to general Ca_V_ channel blockers Cd^2+^ and Ni^2+^ and the selective peptide blockers ω-conotoxin-GVIA and ω-agatoxin-IVA

**Figure 7:**
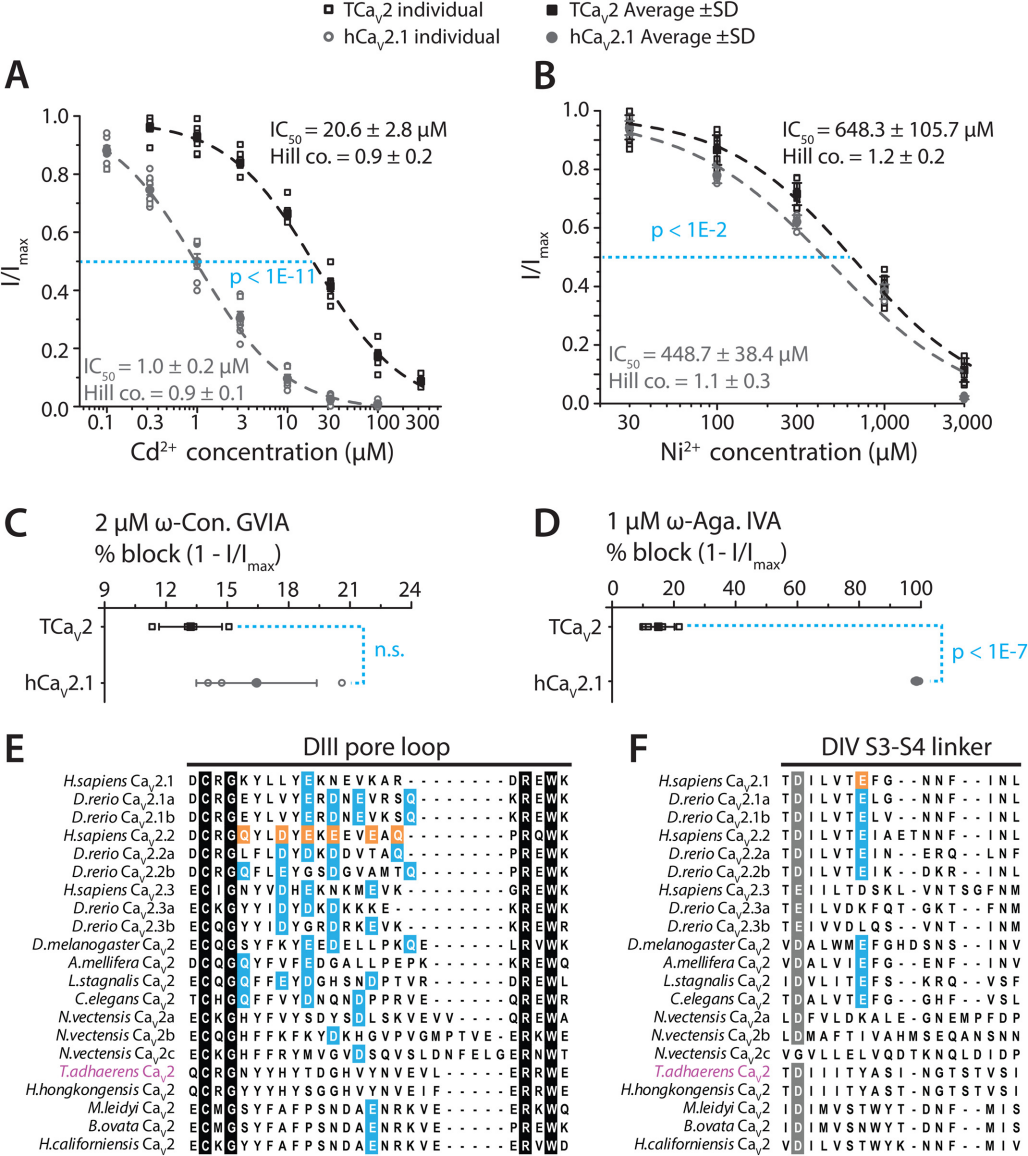
**TCa_V_2 is relatively insensitive to block by divalent metal cations Cd^2+^ and Ni^2+^ and Ca_V_2 isotype–specific peptide toxins** ω**-conotoxin GVIA and** ω**-agatoxin IVA.***A*, dose-response curve for block of peak macroscopic currents of TCa_V_2 and hCa_V_2.1 with increasing concentrations of perfused external Cd^2+^ ions. TCa_V_2 is significantly less sensitive to Cd^2+^ block than hCa_V_2.1, with a roughly 19-fold higher IC_50_ (*p* < 1E−11 using a two-tailed test). *B*, dose-response curves for peak current block of TCa_V_2 and hCa_V_2.1 with increasing concentrations of perfused extracellular Ni^2+^ ions. TCa_V_2 is slightly less sensitive to Ni^2+^ block than hCa_V_2.1 (*p* < 1E−2 using Mann–Whitney comparison of mean IC_50_ values). *C*, plot of average % block ± S.D. of peak macroscopic currents at 0 mV for TCa_V_2 and hCa_V_2.1 by external application of 2 μm ω-conotoxin GVIA, a Ca_V_2.2-specific blocker. *D*, plot of average % block ± S.D. (*error bars*) of peak currents for TCa_V_2 and hCa_V_2.1 by 1 μm ω-agatoxin IVA, revealing complete (98%) block of the human channel compared with only 15% block for TCa_V_2 (*p* < 0.001 using a two-tailed test). *E*, protein alignment of the DIII pore-loop region of various Ca_V_2 channels reveals presence/absence of key residues important for high-affinity block of Ca_V_2.2 channels by ω-conotoxin GVIA. *Orange back-colored residues* reflect positions determined important for block of mammalian channels (84), whereas *cyan back-colored* residues are either conserved or share similar chemical properties. *F*, protein alignment of the DIV S3-S4 linker containing a key glutamate residue associated with affinity block of Ca_V_2.1 channels in mammals (85) (*orange back color*). Notably, these residues are also conserved in channels that are not highly sensitive to ω-agatoxin-IVA.

Ca_V_1 and Ca_V_2 channels are known for their low micromolar sensitivity to block by the metal cation Cd^2+^ and relative insensitivity to Ni^2+^ compared with Ca_V_3 channels (26, 77). Accordingly, perfusion of external Cd^2+^ ions at increasing concentrations blocked hCa_V_2.1 Ca^2+^ currents with an IC_50_ of 1.0 ± 0.2 μm (Fig. 7*A*), whereas Ni^2+^ required a much stronger concentration, with an IC_50_ of 448.7 ± 38.4 μm (Fig. 7*B*). TCa_V_2 was roughly 1.4-fold less sensitive to Ni^2+^ than hCa_V_2.1 with an IC_50_ of 648.3 ± 105.7 μm (*p* < 1E−2 using a Mann–Whitney *U* test) and 1.9-fold less sensitive than the *Trichoplax* Ca_V_3 channel with a previously reported IC_50_ of 335.0 ± 6.5 μm (mean ± S.E.) (28). Compared with hCa_V_2.1, TCa_V_2 was markedly less sensitive to Cd^2+^, with an IC_50_ of 20.6 ± 2.8 μm (*p* < 1E−11, two-tailed test), and requiring 100–300 μm for complete block compared with only 10–30 μm (Fig. 7*B*). Thus, TCa_V_2 differs from vertebrate high voltage–activated channels in its relative insensitivity to Cd^2+^, making it more similar to the expressed Ca_V_1 and Ca_V_2 channels cloned from the snail *L. stagnalis* (11).

Next, we tested the effects of the cone snail and spider peptide toxins, ω-conotoxin-GVIA and ω-agatoxin-IVA, respectively, on TCa_V_2. For mammalian Ca_V_2 channels, ω-conotoxin-GVIA selectively blocks N-type (Ca_V_2.2) channels (78, 79, 80), whereas ω-agatoxin-IVA selectively blocks P/Q-type (Ca_V_2.1) channels (81, 82). Neither TCa_V_2 nor hCa_V_2.1 were sensitive to 2 μm ω-conotoxin-GVIA, only being blocked by 13.2 ± 1.5% and 16.4 ± 2.9%, respectively (Fig. 7*C*; *p* = 0.1 for two-tailed test). Interestingly, work on zebrafish calcium channels involved in synaptic transmission revealed that both Ca_V_2.2 and Ca_V_2.1 channels were highly sensitive to ω-conotoxin-GVIA and relatively insensitive to ω-agatoxin-IVA, indicating that the specificity of these compounds to select Ca_V_2 channel isotypes is not conserved among vertebrate homologues (83). A key locus for ω-conotoxin-GVIA sensitivity has been identified in the DIII pore-loop of mammalian Ca_V_2.2 channels, which contains an EF-hand structure previously implicated in Ca^2+^ permeation (72). Here, directed mutations were found to severely disrupt blocking affinity to the toxin (84). Alignment of this region revealed that both hCa_V_2.1 and TCa_V_2, which are poorly blocked by ω-conotoxin-GVIA, lack the key residues associated with high sensitivity identified in rat Ca_V_2.2 and conserved in the human orthologue (Fig. 7*E*). In contrast, the Ca_V_2.1 and Ca_V_2.2 channel isotypes from zebrafish, which are highly sensitive, resemble human Ca_V_2.2 in this region with tandem negatively charged glutamate and aspartate residues and a C-terminal glutamine residue.

As expected, 1 μm ω-agatoxin-IVA potently blocked the human Ca_V_2.1 channel (98.5 ± 0.6% block), but this was not the case for TCa_V_2 (15.0 ± 5.2% block) (Fig. 7*D*; *p* < 0.001 for two-tailed test). TCa_V_2 lacks a key glutamate residue in the DIV S3-S4 linker associated with affinity block of Ca_V_2.1 channels in mammals (85). However, the presence of this residue in channels that are not highly sensitive to ω-agatoxin-IVA, including zebrafish Ca_V_2.1 channels, Ca_V_2.2 channels, and protostome invertebrate Ca_V_2 channels, suggests that additional sites are important (Fig. 7*F*).

### TCa_V_2 does not exhibit voltage-dependent G-protein inhibition in vitro

A compelling feature of synaptic Ca_V_2 channels is their voltage-dependent inhibition by G-protein β and γ heterodimers, which enables direct neuromodulatory input mediated by G protein–coupled receptors (GPCRs) on presynaptic Ca^2+^ influx and synaptic transmission (86, 87). Indeed, even simple neural circuits exhibit complex neuromodulation, which permits reconfiguring of fast electrochemical machinery for altered input/output properties of neural circuits (88). Our understanding of how and when fast electrochemical signaling machinery and slow neuromodulatory signaling became integrated is limited, and this represents a fundamental question about nervous system evolution (50, 89). With respect to Ca_V_2 channels, neurotransmitter/ligand activation of select GPCRs activates associated Gβγ dimers, which, in a membrane-delimited manner, bind and inhibit Ca_V_2 channels via a direct interaction with regions of the N terminus, C terminus, and I-II linker (86). G-protein inhibition can be temporarily alleviated by bouts of elevated excitation (90), permitting voltage-dependent facilitation of presynaptic Ca^2+^ influx and exocytosis. Previously, the Ca_V_2 channel cloned from the snail *L. stagnalis* was reported to lack voltage-dependent G-protein inhibition (91), suggesting that this important neuromodulatory process is unique to vertebrates and closely related animals. However, subsequent work in cultured *Lymnaea* neurons revealed that endogenous presynaptic Ca_V_ channel currents could be inhibited by activation of dopamine D2 GPCRs (92) and that G-protein inhibition of calcium currents occurs and can be alleviated by strong depolarizing prepulses (92, 93). This suggests that GPCR signaling and specifically Gβγ modulation of presynaptic Ca_V_2 channels was present in the common ancestor of animals with bilateral symmetry (*i.e.* protostomes and deuterostomes).

Here, we sought to explore whether G-protein inhibition of Ca_V_2 channels might be conserved in placozoans. We note from an ongoing transcriptome study that *Trichoplax* expresses over 656 GPCRs, as well as all core intracellular GPCR signaling machinery, including putative G-protein α_i/o_ subunits associated with G-protein inhibition of Ca_V_2 channels (50, 86, 94). We reasoned that the absence of G-protein inhibition observed for the *Lymnaea* Ca_V_2 channel *in vitro* might have been due to divergence between Gβγ subunits in the human cell line used for the electrophysiology experiments and endogenous Gβγ subunits in isolated *Lymnaea* neurons, as well as the binding sites for Gβγ along intracellular regions of the Ca_V_2 channels. Hence, we searched for G-protein β and γ subunits in the *Trichoplax* transcriptome, identifying four Gγ subunits (Gγ_1–4_), and two Gβ subunits (Gβ_1–2_). Protein alignment and secondary/tertiary structure prediction of the *Trichoplax* Gβ subunits and representative homologues from other animals revealed conserved N-terminal amphipathic helices important for interactions with the Gγ subunit and an array of β strands partitioned into seven tryptophan-aspartate (WD) domain repeats, predicted to fold into a 7-bladed β-propeller configuration (Fig. S4) (95, 96). Also evident is that the *Trichoplax* Gβ_2_ subunit is more divergent from other Gβ subunits compared with Gβ_1_. A similar analysis of the *Trichoplax* Gγ subunits revealed a conserved set of tandem N-terminal α helices, which interact with helices of the Gβ subunit (97), and C-terminal cysteine residues that become isoprenylated for integration of the Gβγ heterodimer into the plasma membrane (Fig. S4). Notably, the *Trichoplax* Gβγ proteins differ at some key amino acid positions important for effector function, as reported in yeast (98), but bear determinant amino acids in the Gβ subunits that are required for interactions with mammalian Ca_V_2.2 channels (99, 100) (*i.e.* Tyr^111^, Asp^153^, and Ser^189^; Fig. S4).

**Figure 8:**
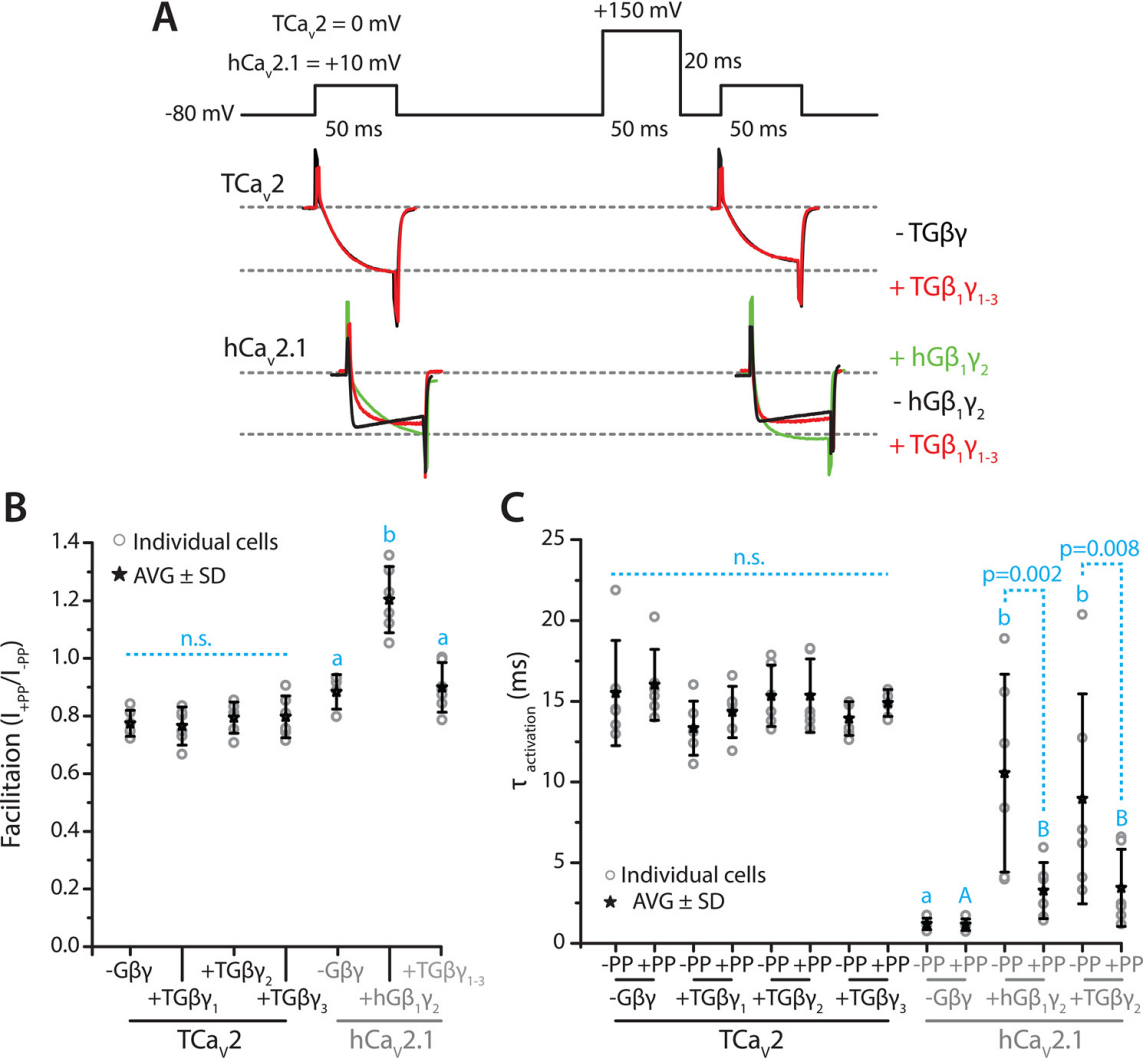
**Absence of voltage-dependent Gβ**γ **inhibition for the TCa_V_2 channel *in vitro*.***A*, illustration of voltage-clamp protocol used to assess G-protein inhibition of TCa_V_2 and hCa_V_2.1 channels *in vitro* (*top*). Sample current traces (normalized to maximal current) elicited by voltage steps to either 0 mV (for TCa_V_2) or +10 mV (for hCa_V_2.1), before and after a strong depolarizing 50-ms prepulse to +150 mV, are show *below*. TCa_V_2 currents did not exhibit facilitation in the presence/absence of co-expressed *Trichoplax* Gβ_1_γ_1–3_ heterodimers. Conversely, in the presence of co-expressed human Gβ_1_γ_2_ heterodimers, hCa_V_2.1 exhibited voltage-dependent facilitation, evident as larger-amplitude currents after the prepulse with faster activation kinetics. Furthermore, the *Trichoplax* Gβ_1_γ_1–3_ subunits caused voltage-dependent facilitation of hCa_V_2.1 activation kinetics, but not current amplitude. *B*, average (AVG) facilitation of peak macroscopic current amplitude after the prepulse ± S.D. (*error bars*) (*i.e.* amplitude after prepulse/amplitude before prepulse). Mean values were not statistically significantly different for TCa_V_2 (denoted as *n.s.*), in contrast to hCa_V_2.1 with a significantly higher amplitude ratio in the presence of human Gβ_1_γ_2_ subunits (*letters above* plotted values denote *p* < 0.001 for paired comparisons using post hoc Holm–Sidak tests after a one-way ANOVA; Table S1). *C*, average τ_activation_ values ± S.D. for monoexponential curves fit over the activation phase of macroscopic currents before and after the 150-mV prepulse. Mean τ_activation_ values were not statistically different for TCa_V_2 (*n.s.*), whereas the prepulse caused acceleration of hCa_V_2.1 activation kinetics in the presence of both human Gβ_1_γ_2_ and *Trichoplax* Gβ_1_γ_1-3_. In the absence of co-transfected G proteins, hCa_V_2.1 activation kinetics were faster than all conditions where G proteins were present and did not significantly change after a prepulse. *Letters above* plotted values denote *p* < 0.001 for paired post hoc Holm–Sidak tests, where *lowercase letters* denote comparisons between the three G-protein conditions without a prepulse, and *capital letters* denote comparisons between the three G-protein conditions with a prepulse (after a two-way ANOVA; Table S1). The reported *p* values in the plot are for Holm–Sidak comparisons of τ_activation_ values within G-protein conditions before and after the prepulse.

We sought to clone the *Trichoplax* Gβγ subunits for *in vitro* co-expression with the *Trichoplax* Ca_V_2 channel. PCR amplification from a whole-animal poly(A) cDNA library was successful for three of the four Gγ subunits (Gγ_1–3_), and one of the Gβ subunits (Gβ_1_). Despite repeated attempts, we were unable to amplify the Gγ_4_ and Gβ_2_ subunits, perhaps due to low-level mRNA expression. Thus, the Gγ_1–3_ and Gβ_1_ were cloned in triplicate into the bicistronic mammalian expression vector pIRES2-DsRed2 that, in addition to the cloned G-protein subunit, expresses the DsRed2 red fluorescent protein that permits co-detection of TCa_V_2 and G proteins via green and red fluorescence, respectively. The consensus sequences for the *Trichoplax* G proteins were submitted to NCBI with accession numbers AZJ50981.1 (Gγ_1_), AZJ50982.1 (Gγ_2_), AZJ50983.1 (Gγ_3_), and AZJ50980.1 (Gβ_1_). As a positive control for electrophysiological experiments, we synthesized and cloned the cDNAs for human Gγ_2_ (NM_053064.5) and Gβ_1_ (NM_002074.5) into the pIRES2-DsRed2 vector for co-expression with the human Ca_V_2.1 channel. To assess the occurrence of voltage-dependent G-protein inhibition, we used a protocol comprising a 50-ms test pulse to 0 mV (for TCa_V_2) or +10 mV (hCa_V_2.1), followed by a ±150-mV prepulse for 50 ms, and a subsequent test pulse to capture the changes in current amplitude and/or channel kinetics resulting from the prepulse (Fig. 8*A*). Under these conditions, the human channel exhibited voltage-dependent facilitation of peak current amplitude, with amplitudes increasing roughly 32% after the prepulse (Fig. 8 (*A* and *B*); +PP/−PP ratio = 0.88 ± 0.06 without Gβ_1_γ_2_ and 1.20 ± 0.11 with Gβ_1_γ_2_; *p* < 0.001 for Holm–Sidak test after one-way ANOVA with *p* < 0.001 and *F* = 22.959; Table S1). Human Ca_V_2.1 activation kinetics were also accelerated by a prepulse in the presence of Gβ_1_γ_2_, with τ_activation_ values of 10.55 ± 6.14 ms without a prepulse, compared with 3.26 ± 1.74 ms with a prepulse (*p* = 0.002 for Holm–Sidak test after two-way ANOVA, *p* < 0.001 and *F* = 21.868 for variation due to G proteins, *p* = 0.001 and *F* = 12.646 for variation due to prepulse, *p* = 0.08 and *F* = 2.714 for variation due to G protein × prepulse; Table S1). Instead, in the absence of co-transfected Gβ_1_γ_2_ subunits, the human channel did not exhibit prepulse-dependent acceleration of activation kinetics (τ_activation_ values of 1.20 ± 0.36 and 1.16 ± 0.36 with and without prepulse; Fig. 8*C*), but kinetics were faster compared with all conditions where G proteins were co-expressed (*p* < 0.05 for Holm–Sidak test after two-way ANOVA; Table S1). Hence, as expected, the 150-mV prepulse temporarily relieved G-protein inhibition of the hCa_V_2.1 channel, resulting in larger-amplitude currents and faster activation kinetics (86). In contrast, we did not observe voltage-dependent G-protein inhibition of the TCa_V_2 channel co-expressed with the *Trichoplax* Gβ_1_γ_1–3_ subunits (Fig. 8, *A–C*). Plus/minus prepulse ratios of peak current were statistically indistinguishable, with mean values of 0.77 ± 0.05 (no Gβγ), 0.77 ± 0.07 (Gβ_1_γ_1_), 0.79 ± 0.05 (Gβ_1_γ_2_), and 0.80 ± 0.07 (Gβ_1_γ_3_). Activation kinetics were also unaffected, with τ_activation_ values of 15.52 ± 3.26 ms (−PP, no Gβγ), 16.03 ± 2.20 ms (+PP, no Gβγ), 13.34 ± 1.67 ms (−PP, Gβ_1_γ_1_), 14.34 ± 1.59 ms (+PP, Gβ_1_γ_1_), 15.34 ± 1.90 ms (−PP, Gβ_1_γ_2_), 15.35 ± 2.27 ms (+PP, Gβ_1_γ_2_), 13.94 ± 1.04 ms (−PP, Gβ_1_γ_3_), and 14.90 ± 0.83 ms (+PP, Gβ_1_γ_3_).

Strikingly, co-transfection of the human Ca_V_2.1 channel with the *Trichoplax* G proteins caused slower current activation compared with control conditions (τ_activation_ = 8.96 ± 6.51 *versus* 1.20 ± 0.36 in the absence of co-transfected G proteins, *p* < 0.001 for Holm–Sidak test after two-way ANOVA; Table S1; Fig. 8*A*). This inhibition was voltage-dependent and could be relieved with a depolarizing prepulse, as observed with human G proteins, decreasing from 8.96 ± 6.51 to 3.44 ± 2.40 (Fig. 8*C*; *p* = 0.008 for Holm–Sidak test after two-way ANOVA; Table S1). However, unlike the human G proteins, the *Trichoplax* homologues did not elicit voltage-dependent inhibition/facilitation of peak current amplitude (+PP/−PP ratio of 0.90 ± 0.09 *versus* 0.88 ± 0.06 in the absence of G proteins; Fig. 8, *A* and *B*). Altogether, it appears as though the *Trichoplax* Ca_V_2 channel does not exhibit direct Gβγ inhibition, at least under our *in vitro* conditions. Nevertheless, the *Trichoplax* G proteins were able to interact with the human channel to produce voltage-dependent inhibition. This suggests that the adaptive changes required to render Ca_V_2 channels sensitive to G-protein modulation occurred primarily via emergent changes in channel sequence/structure, and perhaps the Ca_V_β subunit (86), that permitted interactions with the Gβγ subunits. Accordingly, TCa_V_2 exhibits considerable sequence divergence from vertebrate Ca_V_2.1 and Ca_V_2.2 channels at cytoplasmic N-terminal and I-II linker regions that are required for interactions with Gβγ (86, 101) (Fig. S1).

## Discussion

### Insights into Trichoplax biology inferred from the TCa_V_2 channel

Placozoans provide a unique opportunity for exploring the evolution of Ca_V_ channel properties and cellular functions, in part because they are the most early-diverging animals to possess Ca_V_1–Ca_V_3 channels (3, 8, 10) and also because of their morphological simplicity, bearing only six cell types distinguishable by ultrastructure (15, 65), and absence of true tissues. Our work here characterizing the functional properties of the Ca_V_2 channel from *T. adhaerens* revealed that despite upwards of 600 million years of divergence, TCa_V_2 conducts high voltage–activated Ca^2+^ currents with similar profiles to those of human Ca_V_2.1 and other cloned Ca_V_2 channels (2, 6), such as the homologues from the snail *L. stagnalis* (37) and the honeybee *Apis mellifera* (102). Previously, we showed that the *Trichoplax* Ca_V_3 channel conducts low voltage–activated Ca^2+^ currents similar to orthologues from other animals (28). Thus, it appears as though the core biophysical features of Ca_V_2 channels that distinguish them from at least Ca_V_3 channels were established very early on during evolution. Given that Ca_V_3 channels predate animals and that Ca_V_1 and Ca_V_2 channels likely evolved from a premetazoan Ca_V_1/2-like channel (3, 8, 10), it is perhaps not surprising that extant *Trichoplax* Ca_V_2 and Ca_V_3 channels retain distinct functional profiles. This is also apparent in phylogenetic and sequence/structural analyses, where TCa_V_2 and TCa_V_3 are more similar to their counterparts in other animals than to each other, retaining all differentiating structures. Specifically, TCa_V_2 bears a conserved AID in the I-II cytoplasmic linker (required for interactions with Ca_V_β), C-terminal pre-IQ and IQ motifs (for interactions with calmodulin), and an EEEE Ca^2+^ selectivity filter motif. TCa_V_3, on the other hand, bears a conserved helix-loop-helix gating brake structure in the I-II linker (in lieu of the AID) and an EEDD selectivity filter motif (28). Less clear are the differences between Ca_V_2 and Ca_V_1 channels, in that they exhibit overlapping biophysical properties and share similar structural features. Perhaps an exception is a deeply conserved α-helical structure in the C terminus of Ca_V_1 channels, involved in interactions with cAMP-dependent protein kinase-anchoring protein 15 (AKAP15), which is required for enhancement of macroscopic calcium current by β-adrenergic receptor (GPCR) signaling (10, 103). Currently, we are conducting a functional characterization of the *Trichoplax* Ca_V_1 channel, which will complete the characterization of the placozoan Ca_V_ channel repertoire. A key comparison here will be of Ca^2+^-dependent inactivation and/or facilitation of the *Trichoplax* Ca_V_1 and Ca_V_2 channels, which is possibly one of the key functional differences between these two channel types. These feedback processes are mediated by Ca^2+^ influx through open channels binding to calmodulin proteins preassociated with C-terminal pre-IQ and IQ motifs, which triggers alterations in channel gating (104). In other words, Ca_V_1 channels tend to exhibit pronounced Ca^2+^-dependent inactivation, whereas Ca_V_2 channels show no to moderate inactivation and, in some cases, Ca^2+^-dependent facilitation (104, 105). Conversely, Ca_V_2 channels are generally more readily inactivated by voltage than Ca_V_1 channels (2). Interestingly, recent work has shown that vertebrate and invertebrate Ca_V_3 channels are also regulated by Ca^2+^/calmodulin, but through structural determinants that are different from those of Ca_V_1 and Ca_V_2 channels (106, 107). Physiologically, the differences observed between Ca_V_1 and Ca_V_2 in this regard become apparent during prolonged bouts of excitation. Here, Ca_V_2 channel activity is more susceptible to membrane voltage, where repeated and strong depolarization causes accumulated inactivation and channel silencing, whereas Ca_V_1 channels are less susceptible to inactivation by voltage and, rather, respond to rising levels of cytoplasmic Ca^2+^ (108). If this key difference was established early on, and perhaps conserved in *Trichoplax*, this could in part explain why the two channels have retained several nonoverlapping cellular functions broadly within animals.

A notable feature of *Trichoplax* and placozoans in general is that, despite our knowledge that they express most genes required for fast neural electrochemical signaling (13, 14, 50, 56), including Ca_V_ channels and voltage-gated Na^+^ and K^+^ channels, we know very little about the presence and function of endogenous electrical activity in these animals. This is in contrast to other early-diverging lineages, such as sponges, ctenophores, and cnidarians, for which extensive electrophysiological data have been acquired (3). A challenge in this respect is that dissociated *Trichoplax* cells are difficult to distinguish, are quite small (roughly 1 μm in diameter), and have apparent extracellular matrices that make patch-clamp and sharp electrode recording difficult. Very recently, a first report of endogenous electrical activity of *Trichoplax* and *H. hongkongensis*, recorded from immobilized whole animals using extracellular electrodes, revealed the presence of action potentials that could be elicited by injection of a depolarizing current (109). Furthermore, extracellular recording of isolated crystal cells, involved in *Trichoplax* gravitactic behavior (22), also revealed busts of action potentials upon stimulation. This study has therefore confirmed that electrogenic genes are indeed active in placozoans and that electrical signaling is likely important for *Trichoplax* cell biology and physiology. Key questions that emerge include: how are electrogenic genes differentially deployed in placozoan cell types, and what is the nature and purpose of electrical activity in these cells? Our work here on TCa_V_2 and previously on the *Trichoplax* Ca_V_3 channel reveal functional properties that only make sense in the context of fast oscillations in membrane voltage (*e.g.* graded and action potentials), consistent with the recent description of action potentials. For example, both channels have voltage properties that would render them inactivated and hence nonfunctional at depolarized membrane voltages, suggesting that cells expressing them must retain negative voltages through membrane shuffling of Na^+^, K^+^, and Cl^−^ ions by pumps and exchangers. The distinct and conserved activation properties of TCa_V_3 and TCa_V_2, the former being low voltage–activated and the latter high voltage–activated, indicate a conserved duality in Ca_V_ channel function in *Trichoplax*. Specifically, TCa_V_3 channels, endowed by their low activation voltages, likely contribute toward regulating membrane excitability and action potential generation, whereas TCa_V_2 channels respond to stronger depolarizing events to elicit Ca^2+^ influx and any downstream consequences. Other voltage properties of TCa_V_2 (*e.g.* the observed window current that represents a constant trickle of cytoplasmic Ca^2+^ influx within a discrete range of membrane voltages) can serve functions in regulating membrane voltage and/or Ca^2+^ signaling (26, 67, 68).

We note that, compared with the hCa_V_2.1 channel, TCa_V_2 is somewhat hyperexcitable, at least out under *in vitro* conditions, in the sense that it is less susceptible to inactivation and more readily activated by depolarization. Of course, observations *in vivo* could be dramatically different, because hCa_V_2.1 is active at temperatures near 37 °C, whereas TCa_V_2 is active at temperatures closer to 24–28 °C (110). Nevertheless, it is apparent that TCa_V_2 does not require a very hyperpolarized resting membrane potential to remain active, showing moderate to minimal inactivation at membrane voltages between −30 and −40 mV compared with hCa_V_2.1. This is in stark contrast to the recently characterized Ca_V_2a channel from the cnidarian *Nematostella vectensis* (Fig. 1*B*), one of three Ca_V_2 channel paralogues that appears to have specialized for stinging cell (cnidocyte) discharge. Expressed in HEK-293T cells, the recombinant channel produced high voltage–activated currents and a very left-shifted inactivation curve, rendering it susceptible to inactivation even at hyperpolarized potentials (111). Like hCa_V_2.1, TCa_V_2 exhibited biphasic recovery from inactivation, with a fast component similar to the human channel but a much slower secondary component. Thus, TCa_V_2 would be more susceptible to accumulated inactivation during bouts of prolonged excitation, resulting in a more substantial decline in Ca^2+^ influx over time. Last, we note that the kinetic properties for activation, inactivation, and deactivation are generally slower for TCa_V_2 compared with hCa_V_2.1, differences that are likely amplified when considering the acceleration of kinetics of hCa_V_2.1 at warm physiological temperatures and the slowing down of kinetics of TCa_V_2 at cooler seawater temperatures. An additional consideration that might further differentiate TCa_V_2 and hCa_V_2.1 *in vivo* is that the *Trichoplax* channel is surrounded by different salt compositions in seawater, including a roughly 5-fold higher external Ca^2+^ concentration. Nevertheless, despite some differences, we note that TCa_V_2 exhibits the core functional features of other Ca_V_2 channels involved in synaptic transmission. This includes a dependence on the accessory subunits Ca_V_β and Ca_V_α_2_δ, where efficient *in vitro* expression required co-expression with the rat subunits Ca_V_β1b and Ca_V_α_2_δ_1_. This is similar to what was observed for the Ca_V_2 channel cloned from the snail (37), suggesting that the molecular determinants for interacting with these subunits (*i.e.* the AID for Ca_V_β and extracellular regions for Ca_V_α_2_δ) are strongly conserved. Here, we did not clone and co-express the *Trichoplax* Ca_V_β or Ca_V_α_2_δ subunit cDNAs; however, we note from our transcriptome work that the animal expresses one Ca_V_β subunit and three Ca_V_α_2_δ subunit genes (50). Future studies will be needed to explore the molecular and functional properties of these divergent Ca_V_ channel accessory subunits.

### Specialization of Ca_V_2 channels for fast synchronous exocytosis

We were unable to identify a high-affinity pharmacological compound to block the TCa_V_2 channel *in vitro* that would facilitate exploration of its contributions to *Trichoplax* cellular physiology and behavior (112). A pertinent question is whether TCa_V_2 and Ca^2+^ influx play a role in regulated secretion, given that the animal expresses all of the necessary machinery, including the SNARE complex and associated genes, the exocytosis Ca^2+^ sensors synaptotagmin and complexin, and an array of “neuropeptides” that actively modulate *Trichoplax* motile behavior (10, 13, 14, 15, 50, 56). Based on ultrastructural studies, *Trichoplax* cells contain both dense core and pale vesicles (15, 23), suggesting that like other animals, they can secrete both peptide and small-molecule transmitters, respectively. However, the absence of highly clustered vesicles along the cell membrane, as occurs in the synapse active zone, suggests that *Trichoplax* cells do not carry out robust, synchronous secretion akin to that at the nerve terminal (15, 23). Instead, Ca^2+^-dependent secretion in *Trichoplax* might be more similar to asynchronous, neuroendocrine secretion. In this regard, if co-expressed, there could be complementary contributions from Ca_V_1 and/or Ca_V_3 as occurs in neuroendocrine cells, which, in the case of Ca_V_3, would permit graded subthreshold exocytosis (113).

A key consideration regarding the role of Ca_V_2 and other Ca_V_ channels in driving exocytosis in *Trichoplax* is the proximity of the channels to the exocytotic machinery. This is because the presynaptic calcium sensors that trigger vesicle fusion require substantial increases in cytoplasmic Ca^2+^ concentration (114, 115), which, on a global scale, would lead to cellular toxicity (1). Instead, cytoplasmic Ca^2+^ plumes from open Ca_V_ channels are spatially restricted by rapid removal via Ca^2+^ pumps, exchangers, and chelation agents, resulting in regions near the channel pore of only 20–100 nanometers where Ca^2+^ concentrations reach appreciable levels (*i.e.* Ca^2+^ nanodomains) (35). In synapses where Ca_V_2 nanodomains are positioned very close to vesicles (*i.e.* “nanodomain coupling”), excitation-secretion coupling is thought to be more efficient and to require less total Ca^2+^ than synapses where the channels are further away (35, 36, 116). When channels are positioned further, the probability of release and fidelity declines (35, 114) toward a configuration referred to as microdomain coupling. At microdomain synapses, plumes of Ca^2+^ from separate open channels are thought to sum into larger plumes, where they collectively saturate vesicular calcium sensors of fusion-ready vesicles located in the vicinity (117). An advantage of microdomain synapses is that they are capable of activity-dependent facilitation, where repetitive bouts of excitation, such as trains of action potentials, lead to incremental rises in cytoplasmic Ca^2+^ and a nonlinear increase in the probability of release (35).

Given its ubiquity (1), it is likely that cytoplasmic Ca^2+^ sequestration is also active in *Trichoplax*. Hence, should Ca_V_ channels indeed be driving exocytosis, they must be positioned somewhat close to vesicles, perhaps comprising functional modules held together by specific protein-protein interactions. This would be consistent with the proposal that physical coupling between Ca_V_2 channels and one or more vesicles creates a functional module that can be aggregated at synapses but also deployed more sparsely for nonsynaptic exocytosis (118), as is likely to be the case in *Trichoplax*. Conceivably then, evolution of the presynaptic terminal involved a proteomic aggregation of Ca_V_2 channel-vesicle functional modules, permitting fast, synchronous secretion. Worth noting is that immunolocalization of TCa_V_2 in WGA-positive cells, which co-express the secretory endomorphin-like peptide (18, 23), revealed clustered expression along the outward-facing edge of cells (Fig. 3*A*, *inset*), perhaps representing regions for vesicle fusion. However, the nature of the required apposition between Ca_V_ channels and vesicles would be unclear, because different values of proximity are functional and known to exist (*i.e.* nanodomain *versus* microdomain) (35, 117). Something that confounds this matter further is that the molecular underpinnings that differentiate nanodomain *versus* microdomain arrangements are not entirely clear, and in many synapses, there appears to be a developmental shift from a microdomain configuration to nanodomain (35, 117, 119). We also have a limited understanding of how, and along which animal lineages, these various presynaptic arrangements evolved.

Previously, we noted that the TCa_V_2 channel lacks an acidic amino acid motif at its extreme C terminus with a consensus sequence of (D/E)(D/E/H)WC-COOH, which is conserved in cnidarian and bilaterian Ca_V_2 channels and essential for interactions with the PDZ domains of presynaptic scaffolding proteins Mint (38, 120, 121) and RIM (49, 122, 123). TCa_V_2 also lacks additional C-terminal motifs, upstream of the extreme C terminus, associated with Ca_V_2 channel presynaptic localization and/or function (10, 124, 125, 126). With respect to the Ca^2+^ nanodomain arrangement, RIM has received considerable attention, because it has the capacity to directly interact with Ca_V_2 channels and the vesicular protein Rab3 (127), and its genetic deletion in both vertebrates and invertebrates causes reduced localization of Ca_V_2 channels at the synapse active zone and disrupted synaptic transmission (36, 122, 123, 128). Although *Trichoplax* possesses a RIM homologue, the gene lacks a PDZ domain (10), and in conjunction with the absence of a Ca_V_2 channel (D/E)(D/E/H)WC-COOH motif, it is unlikely that TCa_V_2 is incorporated into homologous RIM-associated proteomic complexes, as reported in animals with synapses. However, this does not preclude the possibility that other redundant presynaptic interactions are present and conserved, where, for example, RIM can interact with the calcium channel Ca_V_β subunit (129) and another Ca_V_2 channel-binding protein, RIM-BP (36). Furthermore, additional interactions that operate independently of RIM (124, 125, 126) could also be conserved, at motifs that are not immediately detectable in protein alignments due to rapid divergence in ligand specificity, as reported for ligands of Src Homology 3 domains (130).

Interestingly, we recently discovered that *Trichoplax* possesses a second class of RIM homologues (dubbed RIM-II), which does bear a PDZ domain but with differences in key regions that suggests different ligand specificity compared with the canonical RIM (*i.e.* RIM-I) (10). RIM-II is broadly conserved in animals, present even in chordates, but was lost multiple times independently, including in vertebrates. Notably, ctenophores, proposed to have independently evolved the synapse (131), have RIM-II and lack RIM-I, making them the only animals with synapses to not have a RIM-I homologue. Whether RIM-II functions at the synapse is not known; however it is expressed in neurons and neuroendocrine cells in the snail, consistent with a role in secretion (10). Future work exploring the proteomic interactions and subcellular localization of the *Trichoplax* Ca_V_2 channel will help clarify its positioning relative to the exocytotic machinery and the homology of protein complexes for its localization.

Indirect evidence that *Trichoplax* is capable of regulated secretion comes from studies on neuropeptide homologues and the small-molecule transmitter glycine that, when applied ectopically, elicit behaviors that emulate those observed naturally. For example, *Trichoplax* expresses endomorphin-like peptide in secretory cells that line the edge of the flat, disc-shaped animal, and ectopic application of this peptide causes *Trichoplax* to stop moving via cessation of ciliary beating on its ventral epithelium (18). Other compounds, also expressed in secretory cells at various anatomical locations, can similarly alter *Trichoplax* behavior, including increased rotation, flattening, or crinkling/writhing into a ball (24, 132). More recently, ectopic application of the small-molecule transmitter glycine was found to elicit concentration-dependent effects on *Trichoplax* behavior, with increased frequency of ciliary beating occurring at low (micromolar) concentrations and whole-body contractions at millimolar concentrations (21). Altogether, these various observations suggest that these compounds are causative agents that underlie changes in *Trichoplax* behavior and, by extension, that their secretion must occur in a regulated fashion such that behaviors can be coordinated.

Here, using a rigorously verified custom antibody, we show that the Ca_V_2 channel is expressed in type-II gland cells also known to express the endomorphin-like peptide and mucous-bearing vesicles that stain with WGA (23). TCa_V_2 was also expressed in other cells along the outer edge of the dorsal epithelium, in areas consistent with other peptide-expressing cells (24). Future work will involve determining the cellular co-expression of the three *Trichoplax* Ca_V_ channels, to provide a framework for appreciating the complementary and differential contributions of the different channels to cellular physiology. Previously, we documented that the TCa_V_3 channel is also expressed in cells along the periphery of the animal (28). However, because both antibodies were generated in rabbits, co-localization of the TCa_V_2 and TCa_V_3 has not been performed; nor has co-localization with TCa_V_1. We hope that ongoing generation of custom polyclonal antibodies in rats will permit effective co-localization experiments.

Downstream of the secretion process, questions also remain about the receptors that make cellular communication possible in *Trichoplax*. For both neuropeptides and glycine, the most likely receptors are GPCRs and peptide- or glycine-gated ion channels. Based on genomic work by ourselves and others, *Trichoplax* was found to express over 656 GPCRs, many of which are homologous to known neuropeptide receptors. Additionally, *Trichoplax* expresses an array of intracellular signaling components, including the G-protein βγ subunits sequenced and cloned in this study (14, 50, 56). Inferred from studies done in other early-diverging animals (133, 134), it is likely that some of the regulation of *Trichoplax* behavior by secreted substances occurs through GPCR signaling, in particular processes that are slower and long-lasting, akin to neuromodulation in the nervous system (88). *Trichoplax* also expresses genes for degenerin/ENaC sodium channels that, in molluscs, vertebrates, and cnidarians, can be gated by neuropeptides (135, 136, 137) and are proposed to mediate synaptic transmission in hydra (138). In our ongoing effort to identify peptide-gated channels in *Trichoplax*, we recently reported that one of the 11 known degenerin/ENaC homologues functions as a Na^+^ leak channel sensitive to block by external Ca^2+^ and H^+^ ions (139), whereas others are gated by protons similar to acid-activated channels from vertebrates and other deuterostomes.^3^ Whether some of the *Trichoplax* degenerin/ENaC channels can also be activated by peptides remains to be determined, a capability that would enable much faster and transient peptidergic signaling than GPCRs, playing out over milliseconds compared to seconds or longer. Last, we note that *Trichoplax* also expresses a considerable number of ionotropic glutamate receptors homologous to NMDA/AMPA/kainate receptors (21) that, based on work done in ctenophores (140), are possibly more sensitive to glycine than they are to glutamate. Indeed, continued functional characterization of these various receptors will be of value toward our understanding of cellular communication in *Trichoplax*, in addition to understanding the capacity of Ca_V_2 and other Ca_V_ channels for driving regulated secretion of signaling compounds that target these receptors.

### On the absence of Gβγ modulation of TCa_V_2 in vitro

Arguably, understanding how the nervous system evolved requires a deep understanding not only of the emergence of fast electrical signaling through synapses and neural circuits, but also how slow neuromodulatory processes co-evolved to regulate the fast signaling machinery (50, 89). Even simple neural circuits are subject to extensive and complex neuromodulation, which can alter ion channel properties and synaptic proteins differently in different neurons for changes in excitability, synaptic connectivity, neural circuit function, and, ultimately, behavior (88). Such an integration occurs for presynaptic Ca_V_2 channels, where various transmitters bind their cognate GPCRs to exert modulatory action on the channels via two distinct pathways: 1) a relatively fast pathway, mediated by direct binding of G-protein βγ heterodimers for voltage-dependent inhibition (141) and 2) a slower pathway that involves downstream second messengers and effector enzymes, such as protein kinases A and C, which phosphorylate Ca_V_ channels and their associated proteins to alter their function (142, 143). Generally, binding of Gβγ to Ca_V_2 channels shifts their voltage dependence of activation to more depolarized potentials and causes a slowing down of activation kinetics, leading to reduced macroscopic current and Ca^2+^ influx (86, 143). Strong depolarizations can alleviate Gβγ binding, permitting a temporary relief of neuromodulatory inhibition of presynaptic Ca_V_2 channels, observed for example during bouts of heightened electrical activity, such as action potential bursts. This form of regulation appears conserved between vertebrates and invertebrates, present in neurons isolated from the snail central nervous system (92, 93). Our inability to observe voltage-dependent Gβγ inhibition of the TCa_V_2 channel, co-expressed with cloned *Trichoplax* G-protein subunits, suggests that placozoans lack the capacity for this type of regulation. Considering the absence of synapses in *Trichoplax*, this functional feature might represent a key evolutionary adaptation toward the specialization of Ca_V_2 channel function at the presynaptic terminal.

We note that TCa_V_2 is similar to the expressed Ca_V_2 channel from the snail *L. stagnalis*, in its sequence divergence from vertebrate Ca_V_2 channels within N-terminal and I-II linker regions that are important for direct interactions with Gβγ proteins (Fig. S1). For the snail channel, replacing these regions with corresponding sequences from rat Ca_V_2.2 failed to produce voltage-dependent G-protein inhibition, even after co-expression with rat Gβγ (91), suggesting that additional structural features are required for the interaction. However, whereas the *Lymnaea* channel lacked this capacity *in vitro*, endogenous Ca_V_ channel currents recorded in neurons were reported to exhibit voltage-dependent G-protein inhibition (92, 93). In preparing for our research, we reasoned that the noted inconsistency was due to sequence divergence between the mammalian G proteins used in the *in vitro* studies, *versus* the endogenous G proteins found in *Lymnaea* neurons. Thus, to circumvent this potential problem in our characterization of the TCa_V_2 channel, we cloned the *Trichoplax* G proteins for *in vitro* co-expression. Interestingly, although the *Trichoplax* channel did not exhibit G-protein inhibition, we found that the *Trichoplax* G proteins could elicit voltage-dependent inhibition of the human Ca_V_2.1 channel. This finding suggests that sequence divergence in the G proteins is permissible and, by extension, that the emergence of the modulatory interaction between Ca_V_2 channels and Gβγ proteins occurred mostly through changes in the channel sequence/structure and not in Gβγ. Indeed, although the *Trichoplax* Gβ_1_ subunit used in this study was somewhat divergent from vertebrate and invertebrate homologues at amino acid positions determined as effector sites in yeast studies (98), the protein bears the three amino acids, Tyr^111^, Asp^153^, and Ser^189^, shown to be required for the interaction between Ca_V_2.2 channels and Gβγ proteins in mammals (99, 100) (Fig. S4). By extension, then, one would expect that the *Lymnaea* Ca_V_2 channel should have exhibited G-protein modulation in the presence of mammalian Gβγ proteins (91), especially after insertion of the appropriate Gβγ-binding sites in the N terminus and I-II linker. One plausible explanation for observed inconsistencies is therefore that the *Lymnaea* Ca_V_2 channel and Gβγ proteins co-diverged from the ancestral linage, such that surrogate G-protein subunits from other divergent species cannot adequately interact with the channel to impose voltage-dependent inhibition. Under this scenario, such a divergence did not happen in the vertebrate/mammalian lineage, hence the ability of the *Trichoplax* G proteins to modulate the Ca_V_2.1 channel. Alternatively, and perhaps less likely, the snail Ca_V_2 channel truly lacks direct Gβγ inhibition, and the phenomenon reported in isolated neurons was due to inhibition of endogenous Ca_V_1 channels. To our knowledge, whether invertebrate Ca_V_1 channels exhibit direct G-protein modulation remains unexplored. Clearly, more work needs to be done to understand the evolution of this important form of synaptic regulation of Ca_V_2 channels. Last, although fast Gβγ regulation was not evident in our experiments for TCa_V_2, it is possible that slow GPCR regulation might occur *in vivo* through other GPCR-dependent intracellular signaling pathways. Similar to fast Gβγ inhibition, slow GPCR regulation of Ca_V_2 channels is conserved in the nervous systems of vertebrates and invertebrates (91, 92, 93, 144).

## Experimental procedures

All animal studies were approved by the University of Toronto Research Oversight and Compliance Office.

### Sequencing and cloning of full-length Trichoplax Ca_V_2, Gβ_1_, and Gγ_1–3_ cDNAs for in vitro expression

The protein coding sequences for *Trichoplax* Ca_V_2, Gβ_1_, and Gγ_1–3_ were cloned from cDNA via RT-PCR. For TCa_V_2, gene-specific primers (Table 1) were used to generate cDNA of the N- and C-terminal halves of the channel coding sequence from whole-animal total RNA (isolated with TRI Reagent; Sigma–Aldrich) via RT-PCR using SuperScript IV reverse transcriptase (Thermo Fisher Scientific). The N- and C-terminal halves of the channel were then amplified independently in triplicate via nested PCR with primers listed in Table 1, the secondary primers bearing restriction enzyme sites for direct cloning into the Clontech vector pIRES2-EGFP (SacII-BamHI for N terminus, SacII-XmaI for C terminus). The nested N-terminal primer also contained a Kozak consensus sequence of GCCACCATGG flanking the start codon, required for efficient translation of the channel protein *in vitro* (145). Full-length TCa_V_2 channel constructs were then assembled via excision of the C-terminal fragment with BamHI-XmaI and cloning into the N-terminal pIRES2-EGFP constructs via the same restriction enzymes. Full-length TCa_V_2 coding sequences within three independent plasmids were determined via Sanger sequencing, and the resulting consensus coding sequence was submitted to GenBank^TM^ with accession number MT506972. For cloning into the pEGFP-C1 vector (Clontech), the coding sequence DNA of TCa_V_2 was excised from the pTCa_V_2-IR-EGFP construct with SacII and XmaI and inserted into matching enzyme sites within pEGFP-C1. This resulted in an in-frame fusion of the EGFP protein coding sequence with the N terminus of TCa_V_2. The *Trichoplax* G proteins were cloned into the Clontech pIRES-DsRed2 vector using a similar strategy, but with cDNA generated with an anchored oligo(dT) primer from whole-animal total RNA, and using restriction sites NheI and BamHI encoded within the nested secondary PCR primers (Table 1). The consensus sequences for *Trichoplax* Gβ_1_, and Gγ_1–3_ were submitted to GenBank^TM^ with accession numbers AZJ50980.1 (Gβ1), AZJ50981.1 (Gγ_1_), AZJ50982.1 (Gγ_2_), and AZJ50983.1 (Gγ_3_). Despite repeated attempts, we were unable to amplify the Gβ_2_ and Gγ_4_ subunit cDNAs.

**Table 1:** Sequences of primers used for cloning *Trichoplax* Ca_V_2 and Gβγ cDNAs

Primer name	Sequence (5′–3′)
TCa_V_2-NT_cDNA	CCTTCAAAATTAATTCAATTAAAAATATCCCGG
TCa_V_2-NT_F1	ACGATCATCTTCAATCGTCTCTAATATG
TCa_V_2-NT_F2	AATAAACCGCGGGAGCCACCATGGCGAGCAGCAGTTTTAATTCATCGG
TCa_V_2-NT_R1	TATTCTTAAAACATAATTTAGGACGGGATCTTC
TCa_V_2-NT_R2	TAGGACGGGATCTTCATTCATAGGATCC
TCa_V_2-CT_cDNA	GTTAAAGTCAGATAAATAAAAAAGAGTCATCATATGC
TCa_V_2-CT_F1	GATCGCAGCAATTATTATAAGCAGTGGG
TCa_V_2-CT_F2	AATAAACCGCGGGTGGGCTACTGGCTGTTGAGGATCC
TCa_V_2-CT_R1	GTCATCATATGCTTATAAATAATATCATTTAAACTGC
TCa_V_2-CT_R2	TTATTTCCCGGGCATTTAAACTGCTGTACATTTTGATATG
TGγ_1__F1	CGTTGTTGGACTTTTTTCTTGGACACG
TGγ_1__F2	ATTATAGCTAGCGCCGCCACCATGGCCGGCGATAAAGCG
TGγ_1__R1	CTTGCCAATCATTTTATTCTTTTATAGCAGC
TGγ_1__R2	TATTAAGGATCCTTATAGCAGCGTGCATTTGCTTTTGTCTGC
TGγ_2__F1	GAATTGATCGTTGACTTGATAAAACGCC
TGγ_2__F2	ATTATAGCTAGCGCCGCCACCATGTCCAATCAATCGACCGC
TGγ_2__R1	CCCTGGTGTAATCTAAAAAGATACTGTGG
TGγ_2__R2	TATTAAGGATCCTTACACCAGGGTACAACGACTTTTCTCC
TGγ_3__F1	CAGTTCAGCGCCATCCACTCC
TGγ_3__F2	ATTATAGCTAGCGCCGCCACCATGCCAGCAAGTATTAGCAACG
TGγ_3__R1	GTTGAAGAATGCAATCGACAAGG
TGγ_3__R2	ATATTAGGATCCTTAAATTAAATTACAGACTTTCTTCC
TGγ_4__F1	GTAATTGGCAGCACAAAATACAGCTG
TGγ_4__F2	ATTATAGCTAGCGCCGCCACCATGAACAAATTTCAAGAAGGCC
TGγ_4__R1	AAGAGATAGGTGGTCATGGAGGAC
TGγ_4__R2	TATTAAGGATCCCTATAATATCGAGCAGCCGCCC
TGβ_1__F1	CTTGGACGAAATTGTTGACCACC
TGβ_1__F2	ATATTAGCTAGCGCCGCCACCATGAGTGATTTAGATCAACTCCGAC
TGβ_1__R1	CATGTAATAACGTTATCTAATTCC
TGβ_1__R2	ATATTAGGATCCCTAATTCCAAATCTTCAGTAAACTGTCCC
TGβ_2__F1	ACTGATTCCACCCAAGTTAAGG
TGβ_2__F2	ATATTAGCTAGCGCCGCCACCATGAAAATGGCTGCGAATGGTG
TGβ_2__R1	CATGTTATAATTCATCTTTTCTATGCCC
TGβ_2__R2	ATATTAGGATCCCTATGCCCAAACTTTTACTGTCTGCCC
Anchored oligo(dT)	TTTTTTTTTTTTTTTTTTVN

### In silico sequence analyses and phylogenetic inference

All protein alignments were carried out using default parameters of the sequence alignment program MUSCLE (146), within the molecular evolutionary genetics analysis (MEGA-X) software suite (147). Alignments were visualized and annotated using JalView version 2.11.1.0 (148) and Adobe Illustrator. Accession numbers for all analyzed sequences are provided in Figs. S1 and S3. The Kyte–Doolittle hydrophobicity plot of the TCa_V_2 channel protein was generated using ExPASy ProtScale (149), using a window size of 9 and default parameters. The maximum likelihood phylogenetic tree of Ca_V_2 channel protein sequences was generated from a protein alignment first trimmed with trimAl (150) using a gap threshold of 95%. Inference was done using IQ-Tree (151), with 1,000 ultrafast bootstrap replicates and an identified best fit model of LG+G4 under the Bayesian information criterion.

### In vitro expression of cloned cDNAs and electrophysiological recording

Detailed methods for culture and transfection of HEK-293T cells were described previously (28, 152). For electrophysiological experiments of *in vitro*–expressed TCa_V_2 and hCa_V_2.1, 2 µg of the pTCa_V_2-IR-EGFP construct or 0.25 µg of the pcDNA3.1-hCa_V_2.1 (EFa/47+) plasmid (49) were transiently transfected into cultured cells in 25-cm^2^ vented flasks, along with 1 µg each of rat Ca_V_β1b and Ca_V_α_2_δ_1_ subunit cDNAs cloned into the mammalian expression vector pMT2 (153). For experiments involving G-protein modulation, transfections were carried out using 1 µg of pTCa_V_2-IR-EGFP or 0.25 µg of pcDNA3.1-hCa_V_2.1, 1 µg each of rat Ca_V_β1b and Ca_V_α_2_δ_1_ subunit cDNAs, and 0.5 µg each of relevant Gβ and Gγ subunit cDNAs cloned into the expression vector pIRES2-DsRed2. For experiments involving co-transfection of hCa_V_2.1 with the *Trichoplax* G-protein subunits, 0.5 µg of each of the three Gγ subunits was co-transfected with 1.5 µg of the Gβ_1_ subunit, along with 0.25 µg of Ca_V_2.1 and 1 µg each of rat Ca_V_β1b and Ca_V_α_2_δ_1_ subunit cDNAs. For negative controls lacking co-expressed G-protein subunits, 0.5 µg of empty pIRES2-DsRed2 was included. Transfections were performed using PolyJet transfection reagent (SignaGen Laboratories) according to the manufacturer's instructions for 6 h, after which cells were washed and transferred to a 37 °C incubator. The next day, cells were treated with trypsin (Sigma–Aldrich), plated onto tissue culture–treated 35-mm cell culture dishes (Eppendorf), and incubated at 37 °C overnight. For patch-clamp experiments, culture dishes were washed and then filled with ∼3 ml of appropriate extracellular recoding solution.

Whole-cell patch-clamp recording of macroscopic Ca^2+^ currents was carried out using an extracellular recording solution containing 140 mm tetraethylammonium chloride (TEA-Cl), 2 mm MgCl_2_, 3 mm CaCl_2_, 10 mm glucose, and 10 mm HEPES (pH 7.4 with TEA-OH, 320 mOsm with glucose). Electrodes were filled with pipette solution containing 120 mm CsCl, 1 mm MgCl_2_, 10 mm HEPES, 10 mm EGTA, 4 mm Mg-ATP, and 0.3 mm Li-GTP (pH 7.2 with CsOH, 300 mOsm with glucose). For pharmacology experiments, stock solutions of Cd^2+^, Ni^2+^, and the peptide toxins ω-conotoxin GVIA and ω-agatoxin IVA (Alomone Laboratory) were prepared by dissolving powders in ultrapure water and then diluted to working concentrations with the 3 mm external Ca^2+^ solution. Solutions containing ω-conotoxin GVIA or ω-agatoxin IVA also contained 0.1 mg/ml cytochrome C (Bio Basic), to minimize adsorption of toxins to contacting surfaces. For experiments comparing Ca^2+^ and Ba^2+^ currents, the external solution was modified to 20 mm CaCl_2_ or BaCl_2_, and MgCl_2_ was removed. The internal solution was modified to 0.5 mm EDTA instead of 10 mm EGTA. Unless otherwise indicated, all reagents for electrophysiological saline solutions were obtained from MilliporeSigma and were of >99% purity.

Whole-cell patch voltage-clamp recordings were performed using an Axopatch 200B amplifier and a Digidata 1440A digitizer controlled with pCLAMP 10 software (Molecular Devices). Pipettes were pulled using a Sutter P-1000 micropipette puller from thick-walled borosilicate capillary tubes (1.5-mm outer and 0.86-mm inner diameter, Sutter) and fire polished with a Narishige MF-900 Microforge such that pipette resistance in the bath ranged from 2 to 5 megaohms. Series resistance was not compensated, and only recordings with minimal access resistance and minimal leak currents (*i.e.* <10% of peak inward current) were used for analyses. Recordings were sampled at 10,000 Hz and then filtered offline at 500 Hz and leak-subtracted (baseline adjustment) using the pCLAMP software. For experiments requiring perfusion of external saline solutions, the Valvelink8.2® gravity flow Teflon perfusion system (AutoMate Scientific) was used. Transformation of peak current-voltage data to normalized conductance values was done using the equation, *g*_ion_ = *I*_peak_/*(V*_command_ − *E*_ion_), where *g*_ion_ is the conductance for Ca^2+^ or Ba^2+^ at a given command voltage (*V*_command_), *I*_peak_ is the peak amplitude of the macroscopic inward current, and *E*_ion_ is the Ca^2+^/Ba^2+^ reversal potential determined by linear extrapolation of the ascending components of the current-voltage data. Tau values for quantifying kinetics of channel activation, inactivation, and deactivation were obtained by monoexponential curve fitting of current traces with the pCLAMP software. Tau values for kinetics of channel recovery from inactivation were obtained by fitting biexponential functions on the data using the software package Origin 2016 (OriginLab). IC_50_ and Hill coefficient values for Cd^2+^ and Ni^2+^ dose-response curves were determined by fitting monophasic dose-response curves over the data using Origin 2016. Origin was also used for fitting Boltzmann functions over conductance/activation and inactivation curves to obtain *V*_½_ and *k* slope values. Statistical analyses were carried out using SigmaPlot and Origin 2016.

### Fluorescence imaging and quantification

For quantification of EGFP fluorescence in transfected HEK-293T cells, triplicate transfections were carried out as described above using 2 µg of pEGFP-TCa_V_2 with or without 1 µg of both rat Ca_V_β1b and Ca_V_α_2_δ_1_ subunits. After incubation at 28 °C for 2 days, the cells were imaged with transmitted and fluorescent light at 20x magnification, using a Zeiss AxioCam MRm Rev3 camera mounted on a Zeiss AxioObserver A1 inverted microscope. All micrographs were taken with the Zeiss ZEN Lite software using the same exposure settings. Integrated density of the acquired fluorescence images was measured using ImageJ software (154), and values were normalized against the highest value for all replicate sets, averaged, and plotted.

### Antibody synthesis

Polyclonal anti-TCa_V_2 antibodies were generated in rabbits. The II-III linker of TCa_V_2 (bases 2130–2564, residues 717–862; Fig. S1) was expressed in BL21 (DE3) *E. coli* as a C-terminal His_6_ fusion protein using the Novagen expression vector pET-28b(+). Protein expression was induced with 0.5 mm isopropyl 1-thio-β-d-galactopyranoside for 4 h, and then cells were harvested by centrifugation and sonicated in lysis buffer containing 500 mm NaCl, 20 mm Tris-HCl, and 10% glycerol, pH 7.9. His-tagged recombinant protein was purified by Ni^2+^ affinity chromatography using nickel-nitrilotriacetic acid His-Bind Resin (EMD Millipore) according to the manufacturer's instructions. Purified protein was then dialyzed into PBS containing 137 mm NaCl, 2.7 mm KCl, 10 mm Na_2_HPO_4_, and 1.8 mm KH_2_PO_4_, pH 7.4. Final yields averaged 0.5 mg/ml. For injection into rabbits for antibody production, purified TCa_V_2 II-III linker peptides were emulsified in Freund's adjuvant, complete (first injection with 500 µg of protein) and incomplete (three subsequent injections, 250 µg of protein for the first boost and then 100 µg for subsequent boosts). After injections, rabbit serum was collected and used for Western blotting and immunostaining experiments. All reagents were obtained from MilliporeSigma.

### Western blotting and fluorescence histochemistry

For Western blotting of *Trichoplax* whole-animal protein lysates, ∼600 specimens were directly lysed in 200 μl of chilled lysis buffer composed of 8 m urea, 50 mm ammonium bicarbonate, and a protease inhibitor mixture (MilliporeSigma). Protein lysates of HEK-293T cells ectopically expressing N-terminal EGFP-tagged or untagged TCa_V_2 channels were prepared as described previously (28). In short, the plasmid pEGFP-TCa_V_2 or pTCa_V_2-IREGFP was co-transfected into HEK-293T cells with rat Ca_V_β1b and Ca_V_α_2_δ_1_ subunits as outlined above, and cells were incubated at 28 °C for 2–3 days to boost channel expression (152). Cells were then washed with PBS and lysed with 300 µl of 1% Nonidet P-40 lysis buffer (125 mm NaCl, 50 mm Tris base, 1.5 mm MgCl_2_, 5% glycerol, 1% Nonidet P-40, pH 7.4). Protein lysates (50 mg) were electrophoretically separated on NuPAGE™ 4–12% Bis-Tris protein gels and then transferred onto nitrocellulose membranes with a solution containing 25 mm Bicine, 25 mm Bis-Tris, 1 mm EDTA, pH 7.2. Following transfer, membranes were washed in TBS-T saline (10 mm Tris-Cl, 150 mm NaCl, 0.05% (v/v) Tween 20, pH 7.4) and blocked for 1 h in TBS-T containing 5% nonfat dried skimmed milk powder at room temperature. The membrane was then incubated overnight at 4 °C with either custom rabbit polyclonal anti-TCa_V_2 or commercial mouse monoclonal anti-EGFP antibodies (Cell Signaling Technologies; 1:3,000 and 1:4,000 dilution in 5% milk TBS-T, respectively). For antigen-blocking experiments, antibody was preincubated with immunization antigen in excess (1:5 mass ratio) overnight at 4 °C to confirm that the antibody was recognizing the protein of interest. Membranes were incubated with a horseradish peroxidase–conjugated goat anti-rabbit or goat anti-mouse secondary antibody (Cell Signaling Technology; 1:2,000 in 5% milk TBS-T) for 1 h at room temperature. Blots were incubated in Clarity Western ECL Substrate (Bio-Rad) for 1–10 min and imaged. Paired gels were run for each experiment, one blotted and the other subjected to Coomassie staining to confirm equal protein content among samples. Western blot analyses performed using custom anti-TCa_V_2 antibodies were done using unpurified antibodies (terminal bleed serum) or preimmune serum (1:1,000 dilution for *Trichoplax* lysates and 1:3,000 dilution for HEK-293T lysates). All indicated reagents were obtained from MilliporeSigma. Quantification of bands observed on Western blots was performed using ImageJ, standardized to corresponding total protein on lanes of Coomassie-stained gels. Differences in protein abundance were determined via one-way ANOVA (*p* < 0.001 and *F* = 387.628; Table S1). Total protein abundance was used for normalizing EGFP-TCa_V_2 protein abundance after statistically validating that total protein abundance did not significantly differ between trials (*p* = 0.629, Kruskal–Wallis test).

For fluorescence histochemistry experiments, *Trichoplax* were frozen and freeze-substituted as described previously (15, 28) with a few modifications. Several *Trichoplax* were transferred to a 500-μl drop of artificial seawater (ASW) placed in the center of Fisherbrand^TM^ Superfrost^TM^ Plus slides (Thermo Fisher Scientific) and left to adhere for 15 min. 300 μl of the ASW was removed and replaced with 500 μl of a 1:1 mixture of ASW and 1 m mannitol. The liquid was removed after 5 min, and the slides were plunged into acetone at −80 °C on dry ice and kept overnight. Slides were then transferred into a glass Coplin jar containing methanol with 1.6% paraformaldehyde, where they were held at −20 °C for 2 h then at room temperature for 1 h. Slides were then rehydrated gradually into PBS and blocked for 15 min in blocking buffer (3% goat serum, 2% horse serum, 1% BSA in PBS). Following rehydration, specimens were incubated overnight at 4 °C with anti-TCa_V_2 antibody (terminal bleed serum) diluted 1:1,000 in blocking buffer (negative control lacked anti-TCa_V_2 antibody). These were subsequently incubated for 2–4 h at room temperature in blocking buffer containing a 1:500 dilution of Alexa Fluor 647 goat anti-rabbit secondary antibody (A-21245, Thermo Fisher Scientific). WGA, Alexa Fluor^TM^ 555 conjugate (Themo Fisher Scientific) was also added together with the secondary antibodies at a dilution of 1:200. Slides were then rinsed in PBS and mounted with ProLong Gold Antifade mountant with DAPI (Invitrogen), and fluorescence micrographs were captured using an inverted LSM 880 confocal microscope (Zeiss) and merged using ImageJ software. Three-dimensional rendering of confocal image stacks was done using Volocity Software (Quorum Technologies). The TCa_V_2 antibody staining was abolished upon preincubation of the primary antibody with the antigen in excess (1:5 mass ratio) overnight at 4 °C.

## Data availability

All data are contained in this article with the exception of the gene sequences for the cloned *Trichoplax* cDNAs, which are available on GenBank^TM^ with accession numbers MT506972 for the TCa_V_2 channel, AZJ50980.1 for G_β1_, AZJ50981.1 for Gγ_1_, AZJ50982.1 for Gγ_2_, and AZJ50983.1 for Gγ_3_.

## Footnotes

3 W. Elkhatib and A. Senatore, unpublished observations.
