# Supplementary material for: Conserved biophysical features of the CaV2 presynaptic Ca2+ channel homologue from the early-diverging animal Trichoplax adhaerens
Source: J Biol Chem. 2021 Jan 13;295(52):18553–78. doi: 10.1074/jbc.RA120.015725 (PMC7939481; doi:10.1074/jbc.RA120.015725)
Supplement: Supplementary file 1 [file mmc1.zip › 162923_2_supp_617027_q2m626.pdf]

Conserved biophysical features of the Ca_V2 pre-synaptic Ca^{2+} channel homologue from the early-diverging animal *Trichoplax adhaerens*

Julia Gauberg[1], Salsabil Abdallah[1], Wassim Elkhatib[1], Alicia N. Harracksingh[1], Thomas Piekut[1], Elise F. Stanley[2] and Adriano Senatore[1]*

[1]Department of Biology, University of Toronto Mississauga, 3359 Mississauga Road, Mississauga, ON L5L 1C6, Canada

[2]Laboratory of Synaptic Transmission, The Krembil Institute, 60 Leonard Street, Toronto, ON M5T 2S8, Canada

*Corresponding author: Adriano Senatore

E-mail: adriano.senatore@utoronto.ca

Tel. (905) 569-4322.

Running title: *Properties of a placozoan Ca_V2 voltage-gated Ca^{2+} channel*

Keywords: Voltage-gated Ca^{2+} channels, Ca_V2 pre-synaptic Ca^{2+} channels, *Trichoplax adhaerens*, patch clamp electrophysiology, synapse evolution, Gβγ inhibition, pharmacology

Figures S1-S4: pages 2 to 8

Supplementary Table 1: pages 9 to 11

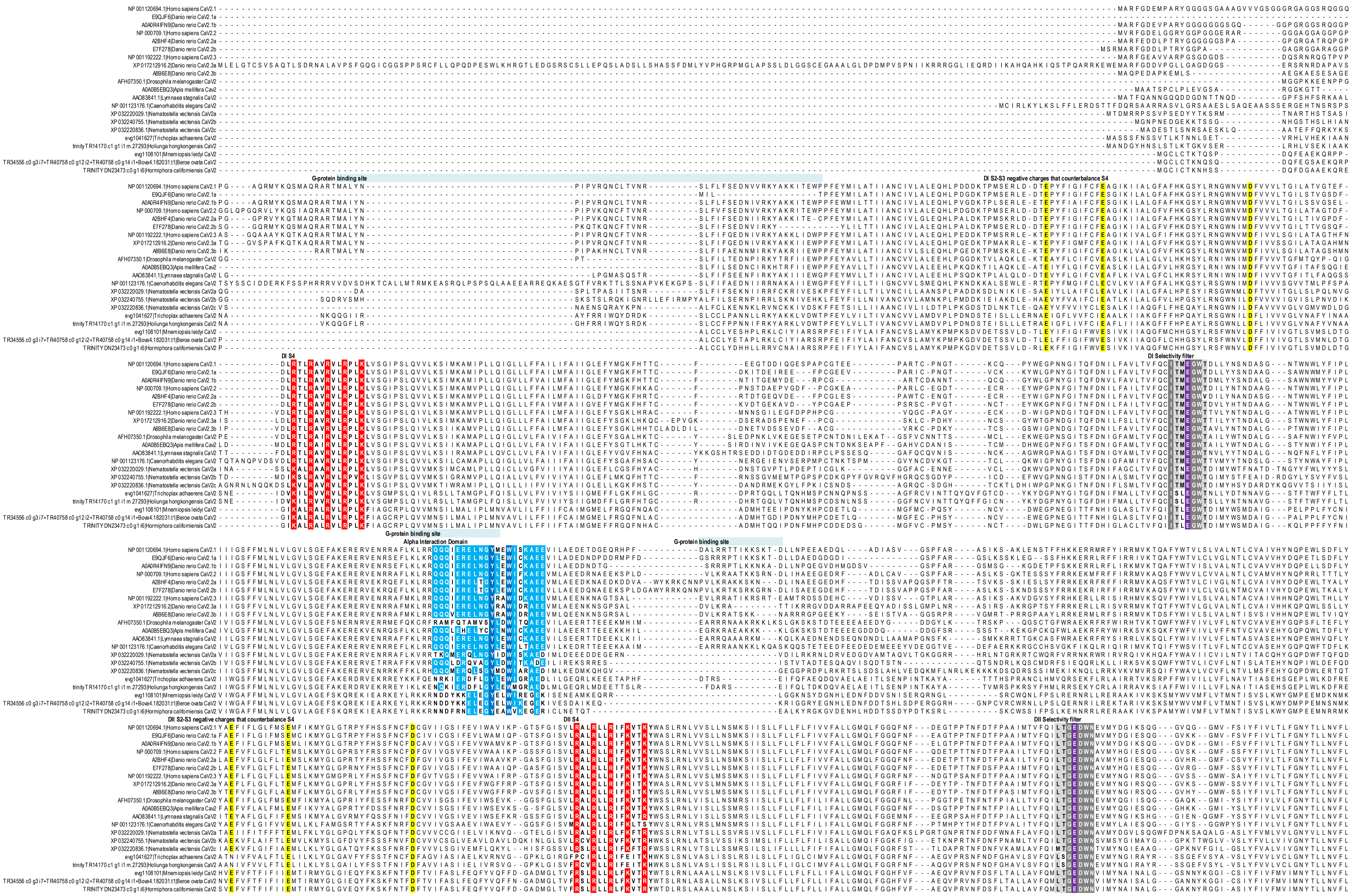

G-protein binding site
DI S2-S3 negative charges that counterbalance S4
DI S4
DI Selectivity filter
G-protein binding site
Alpha Interaction Domain
G-protein binding site
DII S2-S3 negative charges that counterbalance S4
DII S4
DII Selectivity filter

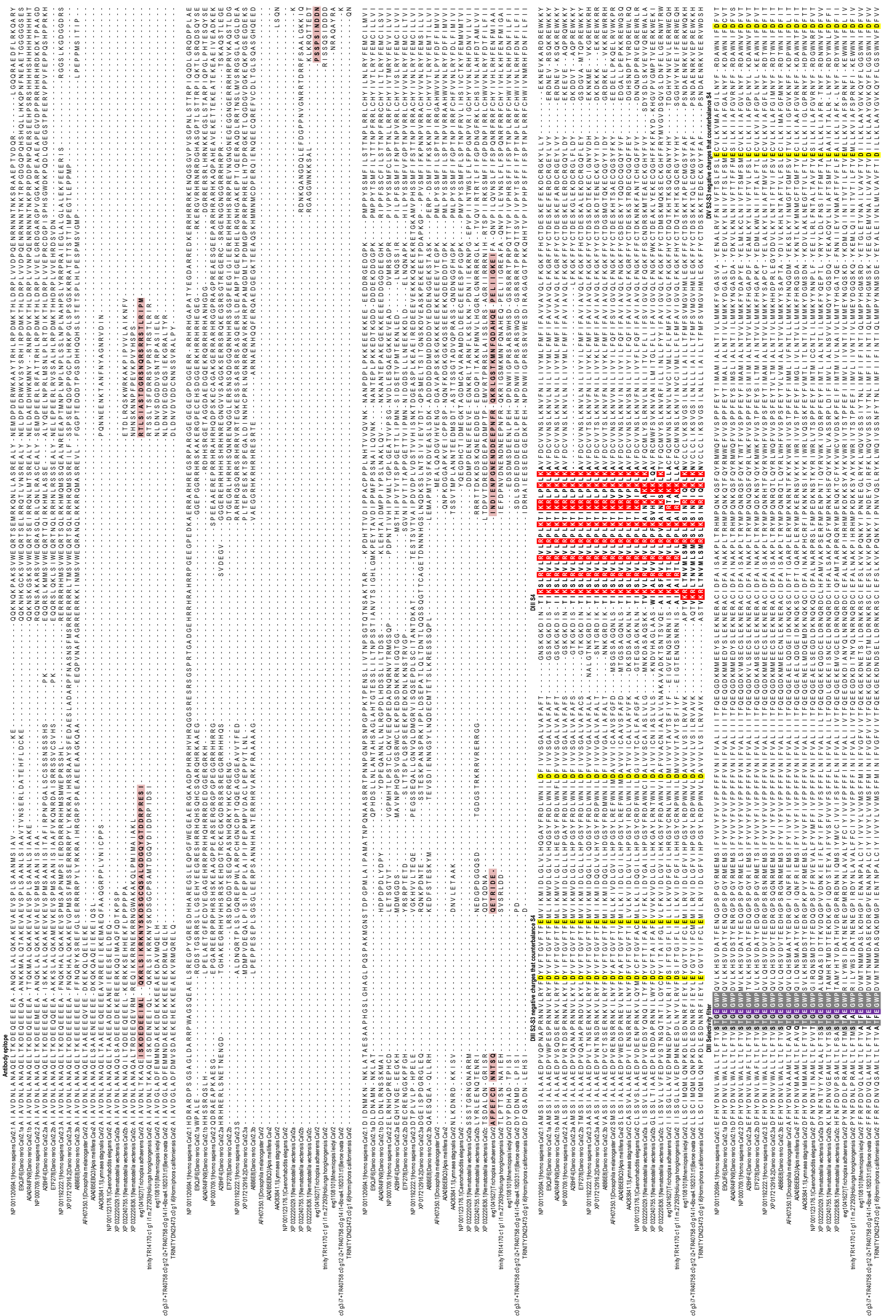

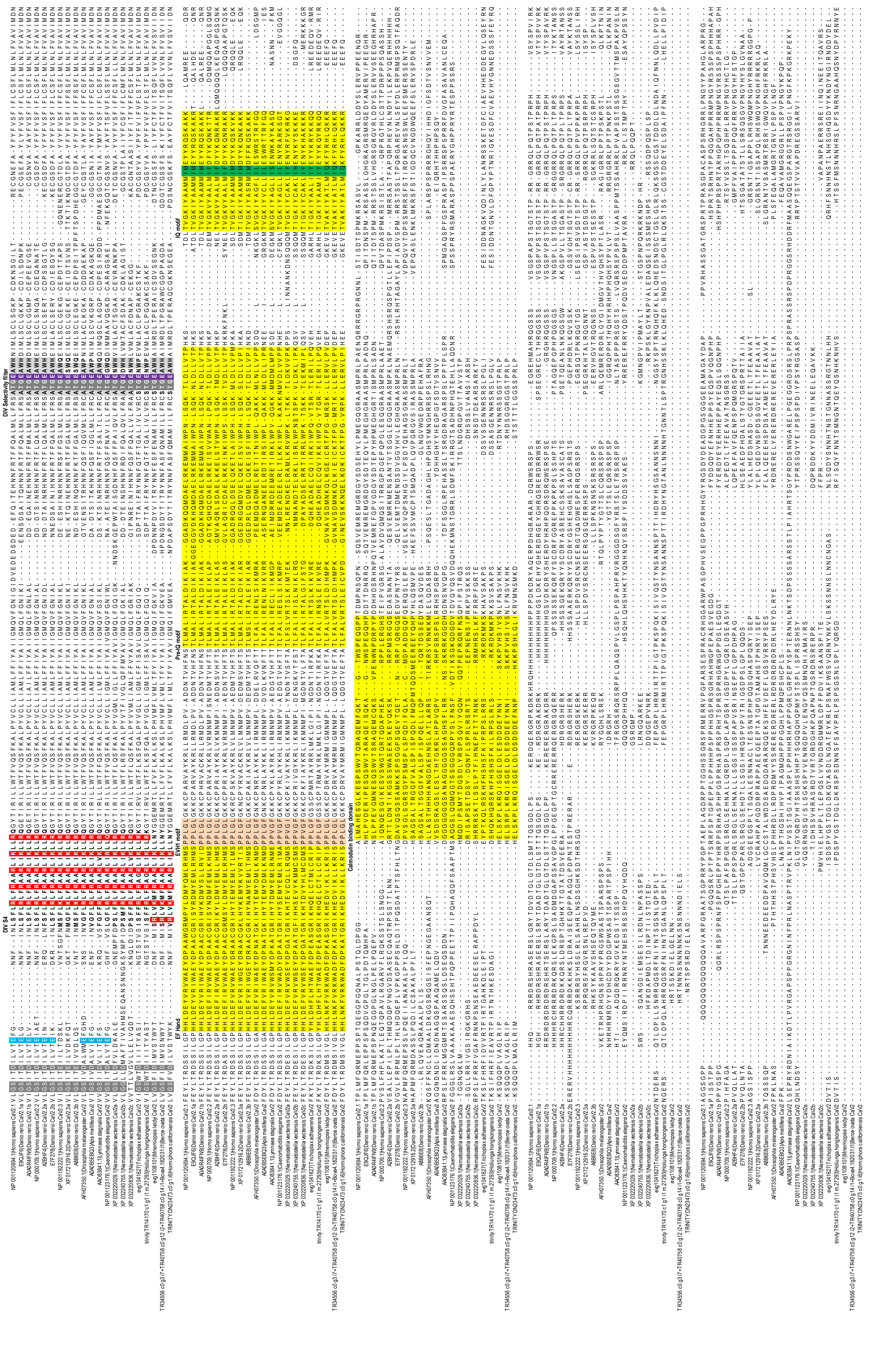
DIV S4
EF hand
EVH1 motif
Pre-IQ motif
IQ motif
DIV Selectivity filter
Calmodulin binding domain

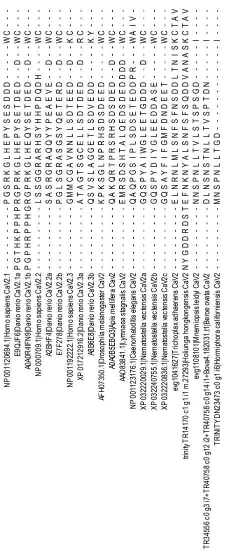

Figure S1. Protein alignment of various Ca$_V$2 channel protein sequences. Key structures and sequence motifs discussed throughout the manuscript are highlighted and labeled.

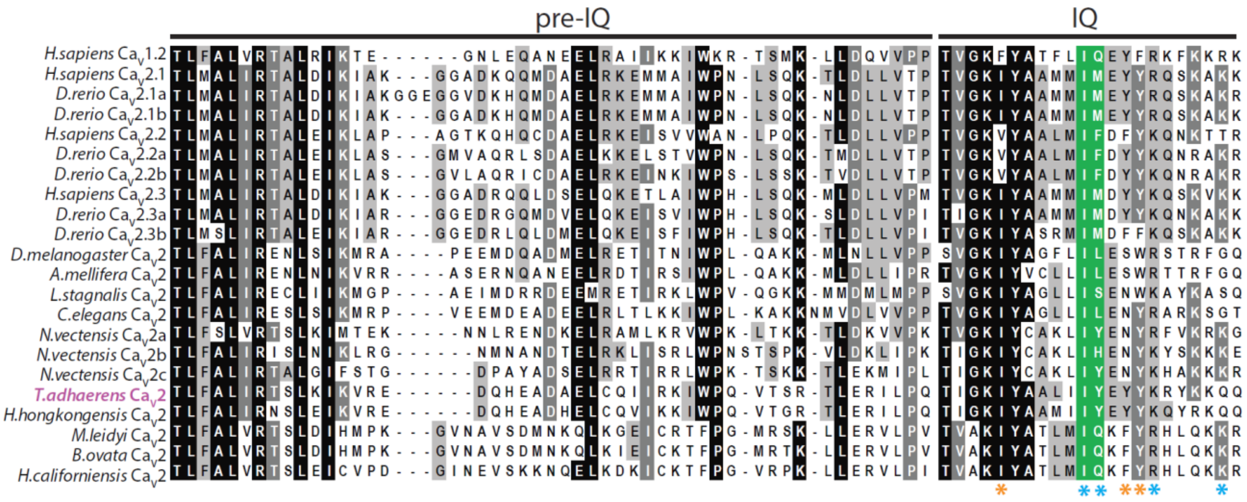

Figure S2. Protein alignment of the pre-IQ and IQ regions within the C-termini of various Ca_V2 channel homologues. The *Trichoplax* Ca_V2 channel IQ region contains key amino acids required for interactions with calmodulin, including an IQ-like motif with consensus sequence [I/L/V]QxxxRxxxx[R/K] (61) (cyan asterisks), flanked by residues that embed within the N- and C-lobes of calmodulin in X-ray crystallography structures (62) (orange asterisks).

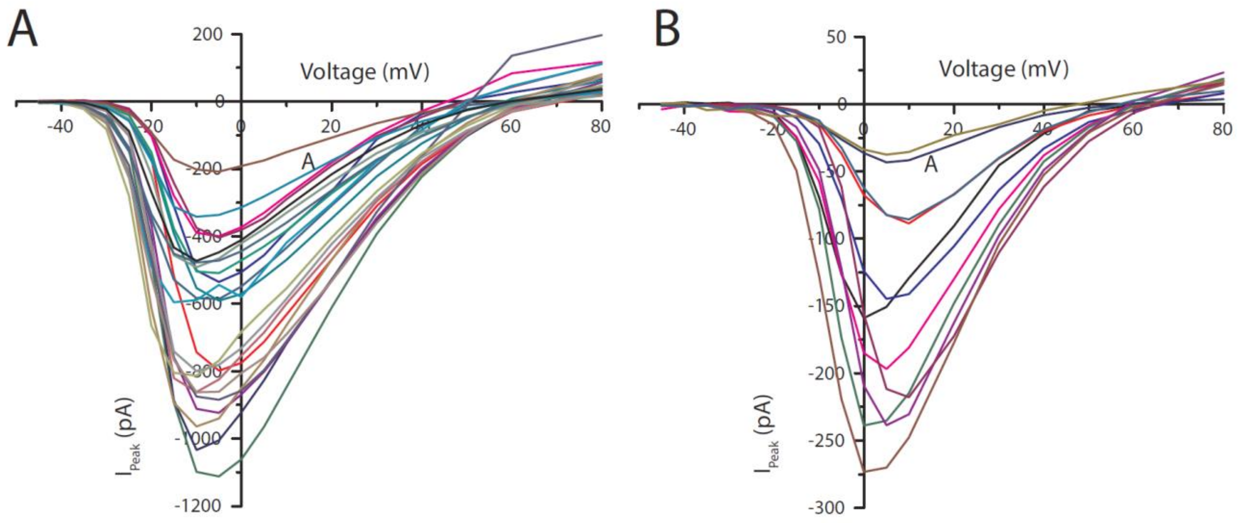

Figure S3. *A*, Un-normalized peak macroscopic currents for TCa_V2 plotted against corresponding voltage steps. *B*, Un-normalized peak macroscopic currents for $hCa_V2.1$ plotted against corresponding voltage steps.

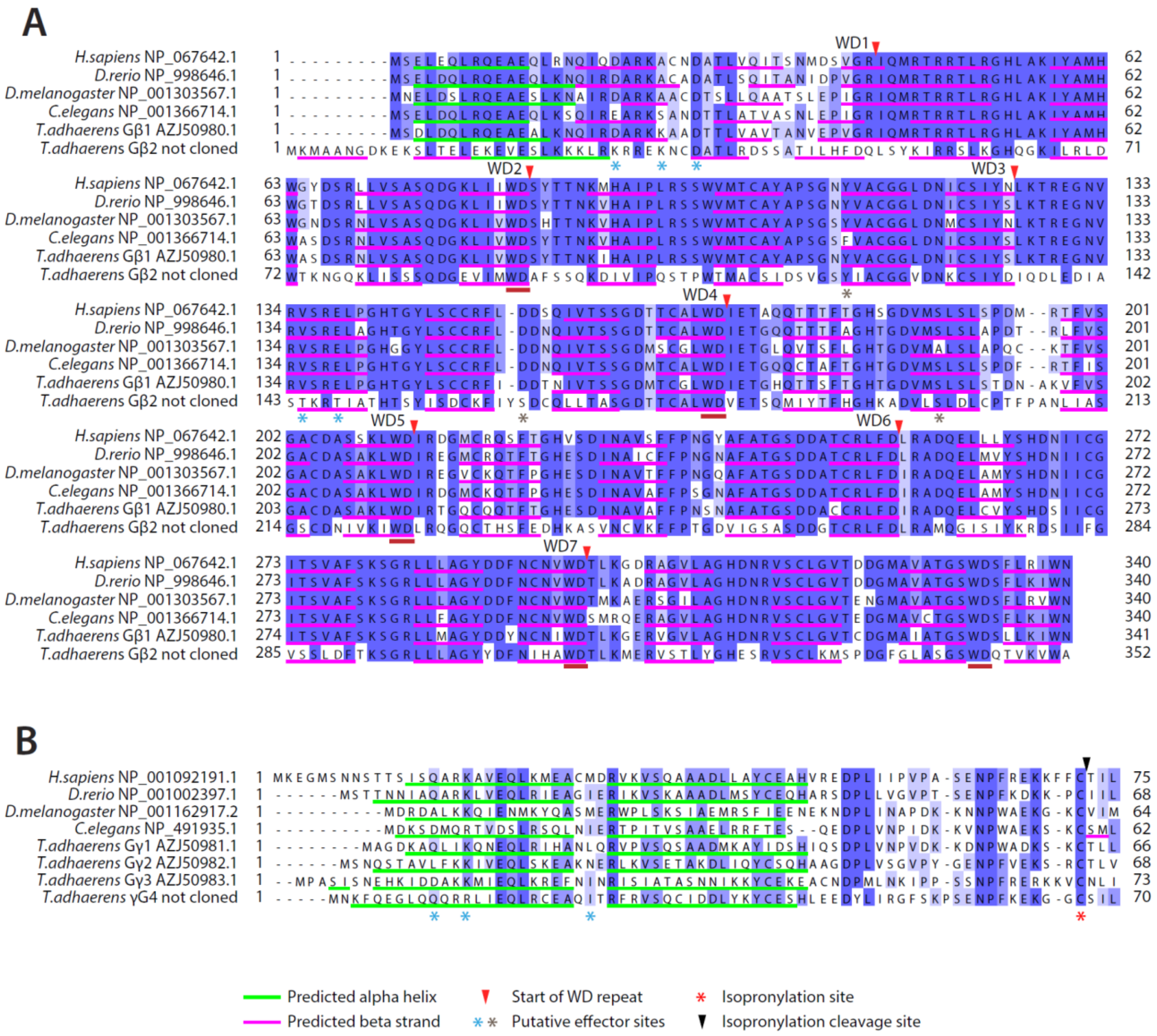

Figure S4. Identified *Trichoplax* G-protein β and γ bear canonical motifs and secondary structures, but vary at key positions associated with effector specificity. *A*, Protein alignment of *Trichoplax* $G\beta_1$ and $G\beta_1$ subunits with representative proteins from other animals. Red chevrons indicate the start positions of WD repeats. *B*, Protein alignment of *Trichoplax* $G\gamma_1$-G γ_4 subunits with representative proteins from other animals. The black chevron indicates putative cleavage sites for isoprenylation of the conserved C-terminal cysteine residue denoted by a red asterisk. Blue asterisks denote amino acid positions identified in yeast as important for effector recognition/function (98), grey asterisks denote determinant amino acids for interaction of Gβγ with $Ca_V2.2$ channels (99,100), green and red underlines represent alpha helices and beta strands predicted with Phyre2 (155), respectively.

Table S1. Metric of statistical analyses reported throughout the manuscript

One-way ANOVA- pEGFP-TCa$_V$2 quantification

Source of Variation	DF	SS	MS	F	P
Between groups	2	13808.071	6904.035	387.628	<0.001
Residual	6	106.866	17.811		
Total	8	13914.937			

Two-Way ANOVA – recovery from inactivation

Source of Variation	DF	SS	MS	F	P
channel	1	1167.700	1167.700	100.274	<0.001
tau	1	2141.438	2141.438	183.891	<0.001
channel x tau	1	1050.104	1050.104	90.175	<0.001
Residual	13	151.387	11.645		
Total	16	4403.374	275.211		

One-way repeated measures ANOVA- activation

TCa$_V$

Source of Variation	DF	SS	MS	F	P
Between Subjects	19	22.583	1.189		
Between Treatments	7	782.174	111.739	158.489	<0.001
Residual	122	86.013	0.705		
Total	148	916.162	6.190		

hCa$_V$2.1

Source of Variation	DF	SS	MS	F	P
Between Subjects	4	0.388	0.0970		
Between Treatments	7	19.004	2.715	99.123	<0.001
Residual	26	0.712	0.0274		
Total	37	20.669	0.559		

One-way repeated measures ANOVA- inactivation

TCa$_V$2

Source of Variation	DF	SS	MS	F	P
Between Subjects	22	110399.952	5018.180		
Between Treatments	6	40031.476	6671.913	30.070	<0.001
Residual	131	29066.072	221.878		
Total	159	182369.247	1146.976		

hCa$_V$2.1

Source of Variation	DF	SS	MS	F	P
Between Subjects	7	7016.574	1002.368		
Between Treatments	6	34990.266	5831.711	14.301	<0.001
Residual	40	16311.045	407.776		
Total	53	57793.257	1090.439		

One-way repeated measures ANOVA- deactivation

TCa$_V$2

Source of Variation	DF	SS	MS	F	P
Between Subjects	8	11.991	1.499		
Between Treatments	9	463.861	51.54	87.22	<0.001
Residual	67	39.592	0.591		
Total	84	526.009	6.262		

hCa$_V$2.1

Source of Variation	DF	SS	MS	F	P
Between Subjects	9	1.83	0.203		
Between Treatments	9	2.016	0.224	15.088	<0.001
Residual	78	1.158	0.0148		
Total	96	5.369	0.559		

Two-Way ANOVA – activation

Source of Variation	DF	SS	MS	F	P
channel	1	730.328	730.328	1142.510	<0.001
voltage	7	280.711	40.102	62.734	<0.001
channel x voltage	7	81.441	11.634	18.201	<0.001
Residual	172	109.948	0.639		
Total	187	1682.978	9.000		

Two-Way ANOVA – inactivation

Source of Variation	DF	SS	MS	F	P
channel	1	3926.923	3926.923	4.824	0.029
voltage	6	63204.286	10534.048	12.942	<0.001
channel x voltage	6	9942.122	1657.020	2.036	0.063
Residual	200	162793.643	813.968		
Total	213	244771.730	1149.163		

Two-Way ANOVA – deactivation

Source of Variation	DF	SS	MS	F	P
channel	1	14.386	14.386	575.305	<0.001
voltage	9	9.45	1.05	41.99	<0.001
channel x voltage	9	1.54	0.171	6.842	<0.001
Residual	132	3.301	0.025		
Total	151	26.522	0.176		

Two-Way ANOVA – Ca^{2+} vs Ba^{2+} $V_{1/2}$

Source of Variation	DF	SS	MS	F	P
ion	1	1019.265	1019.265	244.618	<0.001
channel	1	123.345	123.345	29.602	<0.001
ion x channel	1	14.312	14.312	3.435	0.079
Residual	19	79.169	4.167		
Total	22	1194.066	54.276		

Two-Way ANOVA – Ca^{2+} vs Ba^{2+} slope

Source of Variation	DF	SS	MS	F	P
channel	1	0.814	0.814	1.086	0.310
ion	1	3.535	3.535	4.716	0.043
channel x ion	1	4.559	4.559	6.083	0.023
Residual	19	14.242	0.750		
Total	22	21.338	0.970		

Two-Way ANOVA – $hCa_V2.1$ G-protein Tau

Source of Variation	DF	SS	MS	F	P
G protein	2	2.769	1.3585	21.868	<0.001
Pre-pulse	1	0.801	0.801	12.646	0.001
G protein x pre-pulse	2	0.334	0.172	2.714	0.084
Residual	28	1.773	0.0633		
Total	33	5.785	0.175		

One-Way ANOVA – $hCa_V2.1$ pre-pulse facilitation

Source of Variation	DF	SS	MS	F	P
Between groups	2	0.374	0.187	22.959	<0.001
Residual	14	0.114	0.00814		
Total	16	0.488			
